# Helminth community structure in two species of arctic-breeding waterfowl

**DOI:** 10.1016/j.ijppaw.2016.09.002

**Published:** 2016-09-17

**Authors:** C.L. Amundson, N.J. Traub, A.J. Smith-Herron, P.L. Flint

**Affiliations:** a U.S. Geological Survey, Alaska Science Center, 4210 University Dr., Anchorage, AK, 99508, USA; b Texas Invasive Species Institute, Sam Houston State University, 2424 Sam Houston Ave., Suite B-8, Huntsville, TX, 77341, USA

**Keywords:** *Anser albifrons*, Arctic, *Branta bernicla nigricans*, Climate change, Greater white-fronted goose, Helminth, Pacific black brant, Parasite community

## Abstract

Climate change is occurring rapidly at high latitudes, and subsequent changes in parasite communities may have implications for hosts including wildlife and humans. Waterfowl, in particular, harbor numerous parasites and may facilitate parasite movement across broad geographic areas due to migratory movements. However, little is known about helminth community structure of waterfowl at northern latitudes. We investigated the helminth communities of two avian herbivores that breed at high latitudes, Pacific black brant (*Branta bernicla nigricans*), and greater white-fronted geese (*Anser albifrons*), to examine effects of species, geographic area, age, and sex on helminth species richness, aggregation, prevalence, and intensity. We collected 83 and 58 black brant and white-fronted geese, respectively, from Arctic and Subarctic Alaska July–August 2014. We identified 10 known helminth species (*Amidostomum anseris*, *Amidostomum spatulatum*, *Drepanidotaenia lanceolata*, *Epomidiostomum crami*, *Heterakis dispar*, *Notocotylus attenuatus*, *Tetrameres striata*, *Trichostrongylus tenuis*, *Tschertkovilepis setigera*, and *Wardoides nyrocae*) and 1 previously undescribed trematode. All geese sampled were infected with at least one helminth species. All helminth species identified were present in both age classes and species, providing evidence of transmission at high latitudes and suggesting broad host susceptibility. Also, all but one helminth species were present at both sites, suggesting conditions are suitable for transmission across a large latitudinal/environmental gradient. Our study provides important baseline information on avian parasites that can be used to evaluate the effects of a changing climate on host-parasite distributions.

## Highlights

• We collected two goose species in two high-latitude locations to quantify helminths.
• We identified 10 helminths and 1 previously unidentified trematode in two hosts.
• Waterfowl helminths are readily transmitted in the Arctic and Subarctic.
• Arctic-breeding geese have high helminth prevalence rates and infection intensities.

## Graphical abstract

**Figure:**
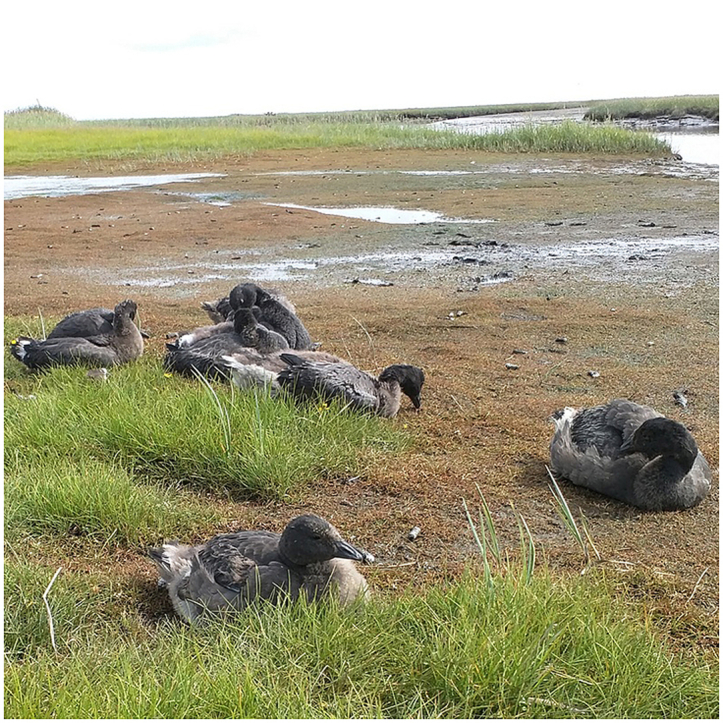

## 1 Introduction

Rapid climate change makes northern latitudes a potential hotspot of change in parasite communities (Polley et al., 2010). In particular, birds host numerous parasites that may negatively affect body condition (Calvete and Estrada, 2003, Souchay et al., 2013), reproduction (Holmstad et al., 2005, Amundson and Arnold, 2010), and survival (Wobeser, 1997). Further, migratory birds are important vehicles for parasite movement across broad geographic areas under suitable environmental conditions and intermediate host availability (Hoberg et al., 2008, Koprivnikar and Leung, 2015). Infected birds may disperse parasites that are now able to complete their life cycles in a warmer Arctic, with implications for other suitable host species and young-of-the-year with naïve immune systems.

Common helminths of birds include nematodes (i.e., round worms), trematodes (i.e., flukes or flatworms), and cestodes (i.e., tapeworms). For most nematode species, definitive hosts are infected through direct uptake of eggs or larvae passed from feces of infected definitive hosts (Cole and Friend, 1999). Conversely, cestodes and trematodes require at least one intermediate host to complete their life cycle. Eggs and larvae passed from a definitive host are consumed by the intermediate host where they develop into an infective stage. Definitive hosts then consume intermediate hosts to complete the cycle (Cole and Friend, 1999). Factors affecting helminth transmission and persistence include: soil and water temperature, rainfall and humidity, and availability of intermediate and definitive hosts (Poulin, 2006, Dudley et al., 2015). Helminth transmission, then, is driven by whether the environment can support different parasite life stages, especially those directly exposed to ambient conditions (e.g., nematode eggs on soil). Thus, parasites and intermediate host populations will likely have complex responses to climate change. Several studies suggest helminth transmission and prevalence will increase with climate change as temperatures 1) change ecological barriers among parasites and hosts (e.g., glacier melt facilitating movement between previously isolated populations of hosts; Kutz et al., 2014), 2) increase growing season length leading to greater transmission opportunity and promotion of intermediate host populations (Plante and Downing, 1989, Elgmork, 2004), and 3) shorten larval development periods (Pietrock and Marcogliese, 2003). However, not all helminths may respond favorably to a warmer Arctic; high temperatures may also decrease helminth transmission (Penner, 1941, Kutz et al., 2014)).

Helminth diversity, prevalence, and infection intensity may vary by host age, sex, and species because of differences in exposure and transmission rates. Host distribution, behavior, habitat use, diet, sexual selection, body size, and immune function all affect exposure and transmission rates among suitable hosts (Gregory et al., 1991). Young birds may have higher exposure to parasites than adults due to different diets (e.g., greater insect consumption in young waterfowl; Street, 1978). Further, young birds often have underdeveloped or naïve immune systems and may be more susceptible to parasitic infection than adults (Cooper and Crites, 1976). Males may have greater helminth burdens than females because of costs associated with testosterone production or developing secondary sex characteristics (e.g., colorful plumage; Poulin, 1996, Hillgarth and Wingfield, 1997, Møller et al., 1999). Additionally, larger birds both among and within species (e.g., adults, males), consume more forage and are thereby more likely to encounter infected intermediate hosts and consume infected feces (Poulin, 1998, Robinson et al., 2008).

Waterfowl, especially, harbor a wide variety of parasites (Ballweber, 2004) usually with unknown implications to hosts. In some cases, helminths have been shown to contribute to negative population-level effects including mass die-offs (Cornwell, 1963, Sandland et al., 2013). Further, studies suggest wide variation in helminth infection dynamics among guilds at high latitudes. Sea ducks that consume mostly marine and benthic invertebrates typically have high helminth diversity (e.g., 31 helminth species; Skirnisson, 2015) and intensity (e.g., 240,000 worms of a single taxon; Galaktionov 1996). Conversely, avian herbivores like geese generally have somewhat lower helminth diversity and infection intensities (e.g., 3–4 cestode species of up to 175 worms; Schiller, 1954); partly because they only opportunistically consume the aquatic invertebrates that typically serve as intermediate hosts (Sedinger and Raveling, 1984, Budeau et al., 1991).

Despite their potential consequences on host populations, very little is known regarding helminth infection characteristics and transmission in waterfowl breeding at high latitudes. Therefore, we investigated the gastrointestinal helminth communities of two avian herbivores, Pacific black brant (hereafter; brant), and greater white-fronted geese (*Anser albifrons*; hereafter white-fronted geese), breeding at two sites in Subarctic (brant) and Arctic (brant and white-fronted geese) Alaska to examine geographic, species, age, and sex variation in helminth species richness, aggregation, prevalence, and intensity. Additionally, we examined whether parasite abundance was associated with host mass to assess one potential consequence of infection.

We examined several theories regarding variation in helminth infection characteristics: First, we hypothesized that shorter growing seasons and lower maximum temperature in the Arctic would reduce diversity and abundance of some helminths through regulation of intermediate host diversity and population size. Second, we expected greater nematode, lower trematode, and similar cestode prevalence and infection rates in brant than white-fronted geese. Colonially-breeding brant forage on primarily well-developed grazing lawns of salt-tolerant sedges such as *Carex subspathacea* or *Puccinellia phryganodes*. For these plants, repeated regrazing results in higher forage quality. Thus, brant frequently forage in previously grazed habitats, which facilitates contact with fecal material, increasing exposure to direct-cycle nematodes. Conversely, non-colonial white-fronted geese feed more on freshwater sedges such as *Carex aquatilis* in a manner that does not promote higher quality forage (i.e., consuming leaf tips and not the entire plant) and thus, are less likely to regraze specific areas. Common trematodes of waterfowl (e.g., Strigeatoidea, Echinostomida; McDonald, 1969) often rely on freshwater gastropods as first and sometimes second intermediate hosts (Sorensen and Minchella, 2001) that are more likely to occur in less saline habitats frequented by white-fronted geese (Ezzat, 1961, Skala et al., 2014). Therefore, we hypothesized white-fronted geese would have greater prevalence and intensity of trematodes than brant. Cestode intermediate hosts are likely copepods or other micro-crustaceans tolerant of salinity (Castro, 1996, Chen et al., 2006) and we did not expect interspecific variation in exposure to cestodes. We also predicted goslings would have lower prevalence rates and helminth diversity than adults because of their smaller body size and lack of exposure to parasites prior to the breeding season (i.e., cross over). However, we predicted goslings would be less immuno-competent and thus suffer higher infection intensities than adults. Lastly, we predicted males would have higher helminth infections and diversity than females because of larger body size and subsequently greater food intake.

## 2 Methods

### 2.1 Study area

**Fig. 1:**
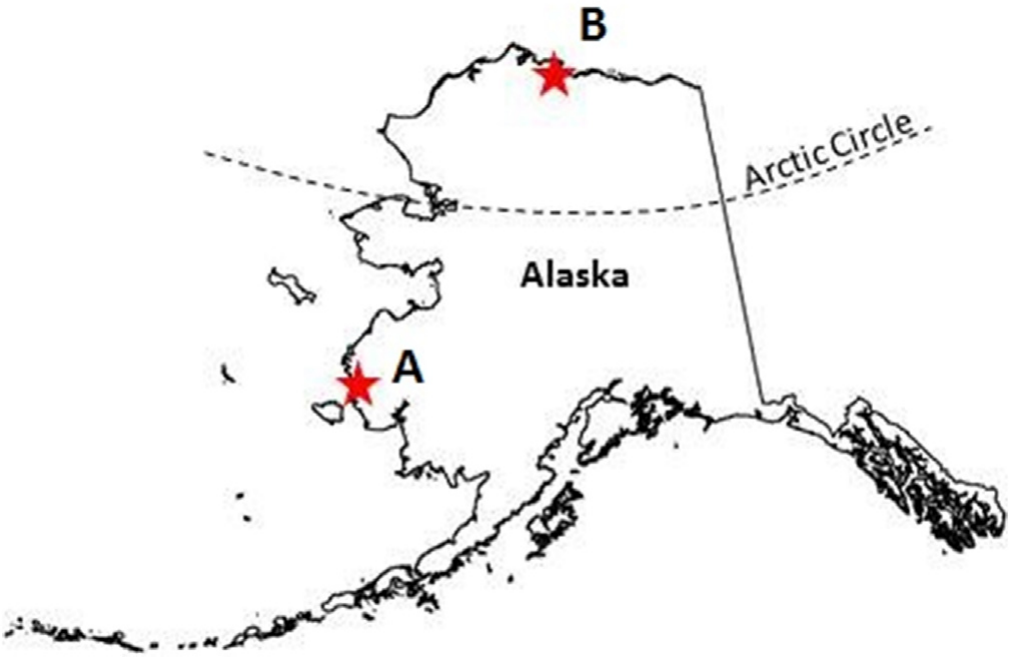
Study sites in Alaska where Pacific black brant and greater white-fronted geese were collected for helminth examination in 2014; A) Yukon-Kuskokwim Delta (61° N 164° W) in Subarctic western Alaska and B) the Arctic Coastal Plain (70° N 154°) in Arctic Alaska.

We collected birds in two important waterfowl breeding areas in Alaska; the Yukon-Kuskokwim Delta (YKD; 61° N 164° W), and the Arctic Coastal Plain (ACP; 70° N 154° W) (Fig. 1). In both areas, waterfowl are distributed along coastal habitat characterized by low elevation short-grass tundra with numerous wetlands. The YKD and ACP vary in breeding season climate; the onset of the growing season is on average 2 weeks later and maximum summer temperatures are ∼7 °C lower on the ACP (Meteoblue v 1.08; Cano-Cruz and López-Orozco, 2015). Waterfowl species composition is similar between the two breeding areas; from 1988 to 2014, 18 of 24 species (75%) observed during aerial surveys were present in both areas (Platte and Stehn, 2015; U.S. Fish and Wildlife Service, unpublished data).

### 2.2 Host species

Both brant and white-fronted geese exhibit bi-parental care and multi-year pair bonds (Baldassarre, 2014). However, the two species vary in size, breeding strategy, habitat use, migratory patterns, and nonbreeding distributions. Brant are colonial breeders and occur in salt-marsh habitats on the coast of both the YKD and ACP (Baldassarre, 2014). Further, brant are Pacific coastal migrants and feed primarily on marine eelgrass (*Zostera* spp.) when in staging and non-breeding areas from southern Alaska to Baja, Mexico. Conversely, white-fronted geese are larger, non-colonial habitat generalists on the YKD and ACP, foraging both in coastal marsh habitats and on freshwater sedges further inland (U.S. Fish and Wildlife Service, unpublished data; Baldassarre, 2014). Arctic-breeding white-fronted geese migrate through the midcontinent and winter in the southern United States largely along the coast of the Gulf of Mexico, and agricultural waste (e.g., corn, rice) is an important food source during the non-breeding season (Baldassarre, 2014). Thus, the two study species are sympatric only during the breeding season on the YKD and ACP.

### 2.3 Sample collection

We collected 42 brant (15 adults, 27 juveniles) from 15 to 21 July 2014 on the YKD, and 41 brant (13 adults, 28 juveniles) and 58 white-fronted geese (16 adults, 42 juveniles) from 31 July to 10 August 2014 on the ACP. We collected geese in conjunction with ongoing mark-recapture studies led by the University of Nevada, Reno, USA and the U.S. Geological Survey. Broods and flightless molting adults were herded into large net pens, and a random sample was euthanized via cervical dislocation. We aged birds as adults or juveniles (i.e., young of the year) via plumage examination and weighed birds (±1 g) using a digital scale. Juveniles were approximately 30 d old at capture (YKD peak hatch date ∼ 17 June, ACP peak hatch date ∼ 7 July; T. Riecke, University of Nevada, Reno, USA and D. Ward and J. Hupp, U.S. Geological Survey, unpublished data). Upon death, each bird was eviscerated from the base of the head to the cloaca, and the heart, lungs, liver, gonads, and kidneys removed for parasite examination. We removed heads just below the lower mandible to examine eyes and nictitating membranes for helminths. Collected tissue was immediately flash frozen in the field using a mixture of 99% ethanol and frozen carbon dioxide (i.e., dry ice) and kept frozen until necropsy (Glass et al., 2002). All collections were authorized by the U.S. Geological Survey Alaska Science Center Institutional Animal Care and Use Committee (Permit # 2014-13) and with permits from the U.S. Fish and Wildlife Service (#MB789758) and the Alaska Department of Fish and Game (#14–092).

#### 2.3.1 Helminth extraction and processing

Before necropsy, samples were thawed at room temperature and divided into functional microhabitats (i.e., anatomical localities within a host) for examination. The following microhabitats were examined for helminths: head; eye surface, nictitating membrane, and tongue and nasal passages; viscera were divided into small intestine, duodenal loop, ceca, proventriculus esophagus, trachea, lungs, heart, and liver. All microhabitats were separated into 1000-ml beakers and flooded with tap water to prevent desiccation of tissues. For gastrointestinal microhabitats, a longitudinal cut exposed the lumen and its contents. The contents were scraped to dislodge attached helminths, and all contents were diluted with additional tap water. The contents of the beaker settled for 5–10 min, and excess (cleared) fluid was drained. The process was repeated several times (depending on the viscosity of luminal contents) until a concentration of helminths remained in clear fluid. Small aliquots of decant (containing helminths) were transferred to petri dishes and the fluid was examined under a dissecting microscope. Non-luminal microhabitats were macerated to dislodge parasites and rinsed into 500-ml beakers and treated as above. Solid residue remaining from each cleared microhabitat was inspected for helminths under a dissecting microscope.

Nematodes were fixed in glacial acetic acid and permanently stored in 7% glycerol. Trematodes and cestodes were fixed in AFA (70% ethanol, formalin, and acidic acid). A sample of 1–2 cestodes and trematodes per species per host were stained in Harris Hematoxylin and counterstained in eosin. Helminth diagnoses were verified through original taxonomic descriptions; Dr. M. Kinsella (HelmWest laboratory, Missoula, Montana, USA) aided in rare species identification. Voucher specimens including those suitable for molecular analyses are available from the Sam Houston State University Natural History Parasite Collection, Huntsville, Texas, USA (see Supplementary Material A).

### 2.4 Statistical measures

Definitions of parasitological terms follow those outlined by Bush et al. (1997). Prevalence represents the number (expressed as a proportion) of hosts infected with a particular parasite species, divided by the number of hosts examined. Intensity (of infection) refers to the number of individuals of a parasite species in a single infected host. Mean intensity is the average intensity of a particular parasite species among the infected members of a particular host species (i.e., total number of parasites of a particular species found in a sample divided by the number of hosts infected with that parasite). Abundance refers to the number of individuals of a parasite species within a single host species examined, including uninfected hosts. Species richness refers to the number of helminth species present within a sample of host species.

Boulinier et al. (1996) was calculated to index, quantify, and estimate helminth aggregation (i.e., the relative amount of clustering of infections among individuals within the population). This index was chosen for its low sensitivity to small sample sizes versus common aggregation indices (e.g., Poulin's D, k; Poulin, 1993, Sherrard-Smith et al., 2015). Confidence intervals for prevalence were calculated using the Sterne (1954) method for binomial distributions (Reiczigel, 2003), and we bootstrapped (n = 1000) confidence intervals (CIs) for mean parasite intensity (Rózsa et al., 2000) and Boulinier's *J* (Sherrard-Smith et al., 2015). We restricted CI estimates for Boulinier's *J* to groups with >10 individuals infected because preliminary analyses suggested this was the minimum necessary to accurately simulate *J*.

Since sampling was not fully factorial (i.e., site × species), we examined sex, age, and either species or site effects including possible interactions on mean intensity and prevalence of combined helminth classes and individual parasite species. Thus, our global model structure was y∼α+Site×Age×Sex+Species×Age×Sex (n_parameters_ = 16). For mean intensity, negative binomial generalized linear models were built to account for excess variance relative to the mean that resulted in poor fit of Poisson regression models. Factors affecting prevalence were evaluated using generalized linear models with a logit link, while factors affecting mass and species richness of individuals were evaluated by Gaussian regression. Species richness was a count, but data were approximately normally distributed around the mean (Fig. B1). Host mass was not included as a covariate in prevalence and intensity analyses because mass varies by age, sex, species, and, possibly, site (Table B1). Therefore, we would have been limited to examining within-subgroup differences in mass, which was precluded by small sample sizes in some subgroups.

We visually examined model residuals to ensure we met distribution assumptions and evaluated model fit of global generalized linear models with a chi-square goodness-of-fit test (Hosmer and Hjort, 2002). We restricted regressions to response variables with >10 birds uninfected and >20 birds infected to ensure sufficient sample size and variability in the data. We considered additive effect-only models for species with >10 but <40 birds infected to avoid overfitting models.

Parameters in regression analyses were selected to best fit the data using Akiake's Information Criterion adjusted for small sample size (AIC_c_). Models evaluating factors affecting unadjusted body mass had age × sex × species interactions fixed, and we evaluated potential effects of age × sex × site because previous studies suggest brant breeding on the YKD may weigh less than brant breeding on the ACP (Ward et al., 2004). We also considered abundance of parasite class or species, and species richness as covariates. Helminth types (e.g., nematodes) were not considered in the same models as individual species within the same taxonomic group as they were often highly correlated. Otherwise, we considered all plausible combinations because all variables and their interactions were biologically reasonable and likely influenced response variables, but we did not know which combination of factors would be most supported (max number of models = 70; Doherty et al., 2010). Main effects were interpreted with variable importance >0.6, and interactions only if they were included in the most supported model (i.e., lowest AIC_c_ value). We recognize equivalent models (i.e., ΔAICc < 2) with one fewer parameter may suggest weak support for the omitted variable (Arnold, 2010). Thus, our inference can be considered conservative. All analyses were completed using the *MuMIn*, *binom*, *vegan*, *psych*, and *MASS* packages in R (v 3.1.1; R Core Team, 2015). Confidence intervals are reported for metrics because ±SD is not informative for aggregated distributions of parasites (Rózsa et al., 2000) and at 85% because they are more consistent with an parameter significance thresholds within an AIC framework (Arnold, 2010).

Differences in Boulinier's *J* were tested among groups for the entire dataset; brant at both sites only, and both host species in the Arctic by comparing CIs. To maintain consistency with significance thresholds used throughout the manuscript, differences in aggregation were interpreted for groups with 85% CIs that do not overlap (Sherrard-Smith et al., 2015).

Correlations among presence/absence data were evaluated using a Pearson's product-moment correlation matrix. Strong correlations in presence among helminth groups may indicate host susceptibility, or parasite species with transmission sites or intermediate hosts in common. We conducted paired t-tests and report associations from test statistics with α < 0.15 (i.e., 85% CI equivalent).

## 3 Results

**Fig. 2:**
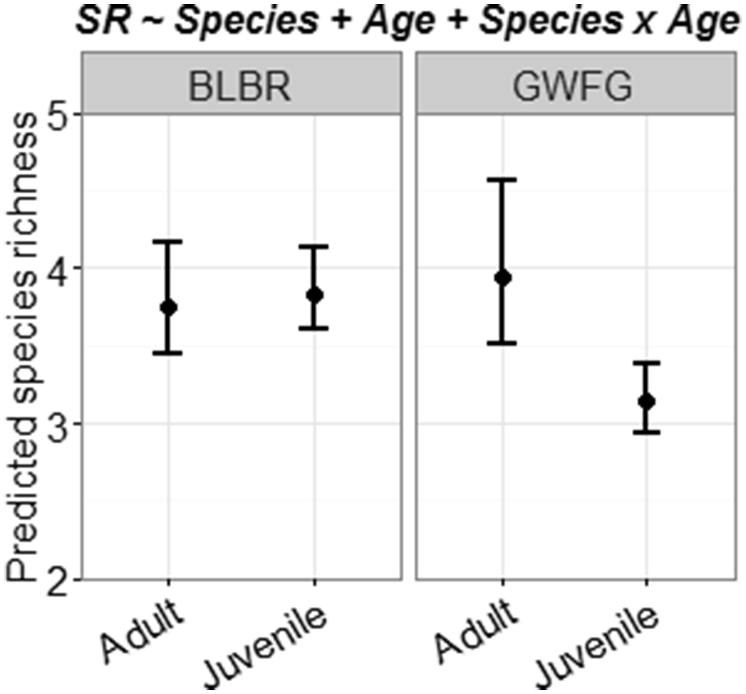
Predicted helminth species richness (SR) for Pacific black brant (BLBR) and greater white-fronted geese (GWFG) collected from Subarctic and Arctic Alaska (2014). Circles represent predicted means and error bars denote 85% confidence intervals. Predictions are based on the most supported model from AIC_c_ selection and all interactions include lower-order effects (see title).

We identified 11 species of helminths (6 nematodes, 3 cestodes, and 2 trematodes) from 141 geese examined, representing 14,364 individual helminths. Helminths occurred in 5 microhabitats (i.e., gizzard, proventriculus, duodenal loop, small intestine, and ceca), of which the small intestine was the most commonly occupied. On average, geese were infected with 3.62 (SD = 1.24) helminth species (range = 1–7) and only 7 birds had a single helminth species present (Fig. B1). Parasite species richness was lower in juvenile white-fronted geese (richness = 3.14, 85% CI: 2.94–3.39) than adult white fronted geese or brant of either age (mean richness = 3.83, 85% CI: 3.54–4.21; Fig. 2). All geese were infected with at least one helminth species and all helminth species were present in both age classes, sexes, species, and sites except the nematode *Epomidiostomum crami*, which was only present in the Arctic, and only present in white-fronted geese with the exception of one roundworm found in a brant (Table B2). The average intensity of infection was 101.23 worms per bird (85% CI: 92.34–114.98, range = 2–659); cestodes numerically dominated the component community for both host species (Table B3).

Virtually all geese were infected with nematodes and cestodes (0.965 and 0.929, respectively), but trematode prevalence was low (0.142; Tables 1 and B4). Relatedly, trematodes (J = 25.80, 85% CI: 13.95–52.38) were more aggregated than cestodes (J = 1.44, 85% CI: 1.11–2.08) or nematodes (J = 1.03, 85% CI: 0.77–1.49), suggesting a few birds had intense trematode burdens, but cestode and nematode infections were more uniform across hosts (Table B5).

Of the 12 possible age × sex × species × site groups, two nematodes (*Trichostrongylus tenuis* and *Heterakis dispar*) and one cestode (*Tschertkovilepis setigera)* were present in all groups (range = 3–12 groups, Table B2). Two nematodes, *Tetrameres striata* and *Amidostomum spatulatum*, were not present in Subarctic or Arctic juvenile brant, respectively. Further, the cestode *Drepanidotaenia lanceolata* was not present in adult white-fronted geese. We observed a previously undescribed trematode in 5 birds, mostly white-fronted geese (Table B4).

### 3.1 Factors affecting prevalence and intensity

We evaluated factors affecting prevalence and intensity of combined trematodes, three nematode species, and three cestode species. We evaluated only additive effects on combined trematode prevalence and intensity because few birds were infected (n = 23). We also evaluated factors affecting combined nematode or cestode intensity, but could not evaluate factors affecting combined cestode or nematode prevalence because so few (only 10 and 6, respectively) hosts were uninfected. However, cestodes were more aggregated in the Subarctic than Arctic (*J* = 3.62 and 1.03, respectively) suggesting patchier distribution of cestode infection among birds (Fig. B2). Further, in Arctic geese, nematodes were more aggregated in white-fronted geese and juveniles than brant and adults (Table B5, Fig. B3).

Model results suggest combined trematode prevalence was similar among sites, age classes, and sexes, but higher in white-fronted geese (0.224, 85% CI: 0.155–0.313) than brant (0.843, 85% CI: 0.496–0.140); intensity was higher for juvenile white fronted-geese (31.49, 85% CI: 19.10–51.91) than adults (8.14, 85% CI: 3.89–17.03) or brant of either age (2.33, 85% CI: 1.11–4.91).

**Fig. 3:**
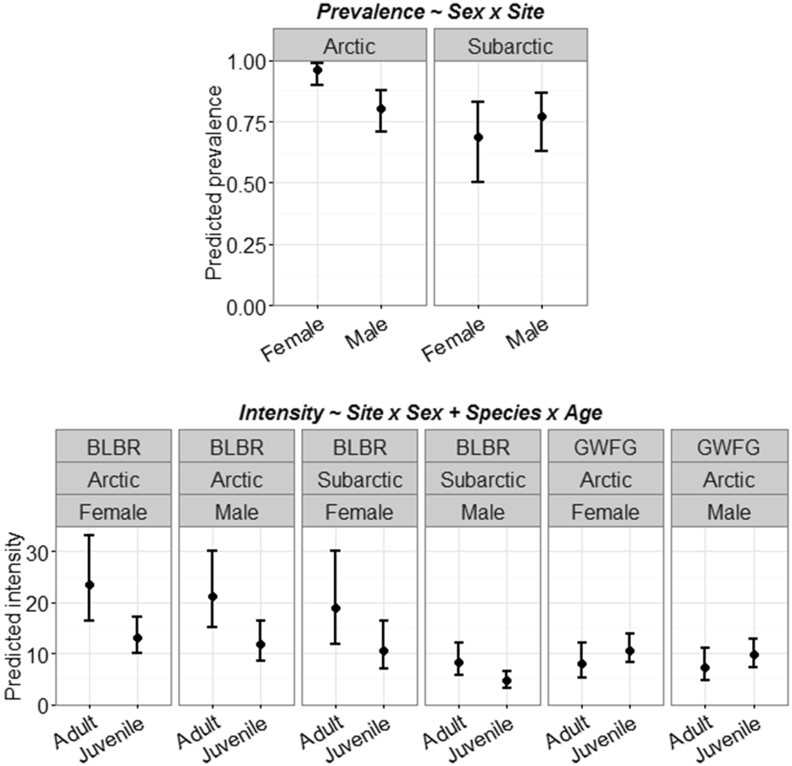
Predicted prevalence (top) and helminth infection intensity (bottom) for the nematode *Trichostrongylus tenuis* enumerated in Pacific black brant (BLBR) and greater white-fronted geese (GWFG) collected in Arctic and Subarctic Alaska (2014). Circles represent predicted means and error bars denote 85% confidence intervals. Predictions are based off the most supported model from AIC_c_ selection and all interactions include lower-order effects (see titles).

**Fig. 4:**
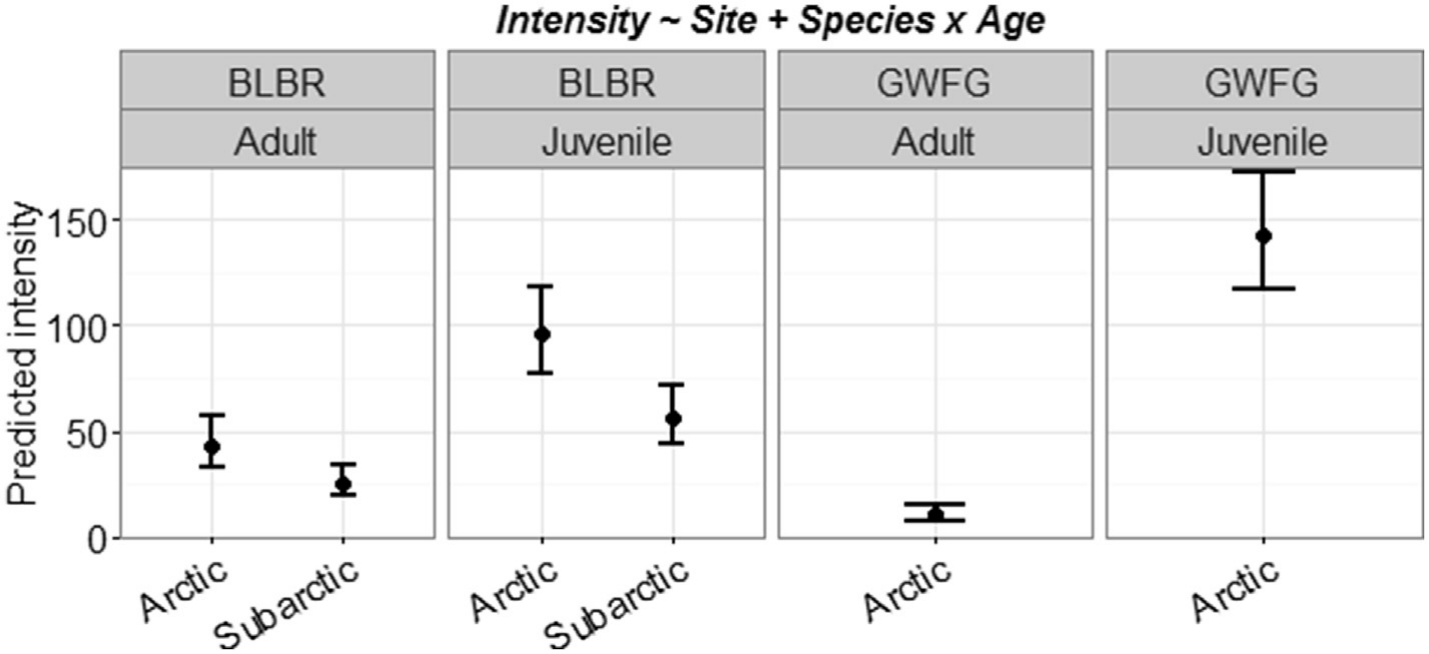
Predicted prevalence (top) and helminth infection intensity (bottom) for the cestode *Drepanidotaenia lanceolata* enumerated in Pacific black brant (BLBR) and greater white-fronted geese (GWFG) collected in Arctic and Subarctic Alaska (2014). Circles represent predicted means and error bars denote 85% confidence intervals. Predictions are based off the most supported model from AIC_c_ selection and all interactions include lower-order effects (see titles).

We detected site effects on nematode prevalence for 2 species; *T. tenuis* had greater prevalence in Arctic females (0.962, 85% CI: 0.90–0.986) than Arctic males (0.804, 85% CI: 0.707–0.875) or Subarctic birds (0.738, 85% CI: 0.582–0.852; Fig. 3), but *A. anseris* had lower prevalence in Arctic brant (0.488; 85% CI: 0.177–0.127) than Subarctic brant (0.333, 85% CI: 0.238–0.445) and white-fronted geese (0.345, 85% CI: 0.261–0.439; Fig. B4). Further, prevalence of the nematode *H. dispar* was higher in juveniles (0.691, 85% CI: 0.612–0.759) and brant (0.843, 85% CI: 0.760–0.902) than adults (0.523, 85% CI: 0.418–0.627) and white-fronted geese (0.345, 85% CI: 0.253–0.455; Fig. B4). Combined nematode infection intensity was higher in adults (29.79, 85% CI: 24.53–36.18) than juveniles (20.60, 85% CI: 18.03–23.54; Fig. 4), but otherwise did not vary by site, species, or sex. Patterns in intensity emerged among the 3 nematode species examined; generally intensity was higher in the Subarctic, adults, and for males (Table B3, Fig. B5). However, *T. tenuis* intensity was lower in Subarctic males (6.11, 85% CI: 4.24–8.80) than Arctic birds of either sex (12.31, 85% CI: 9.07–16.75; Fig. 3).

**Fig. 5:**
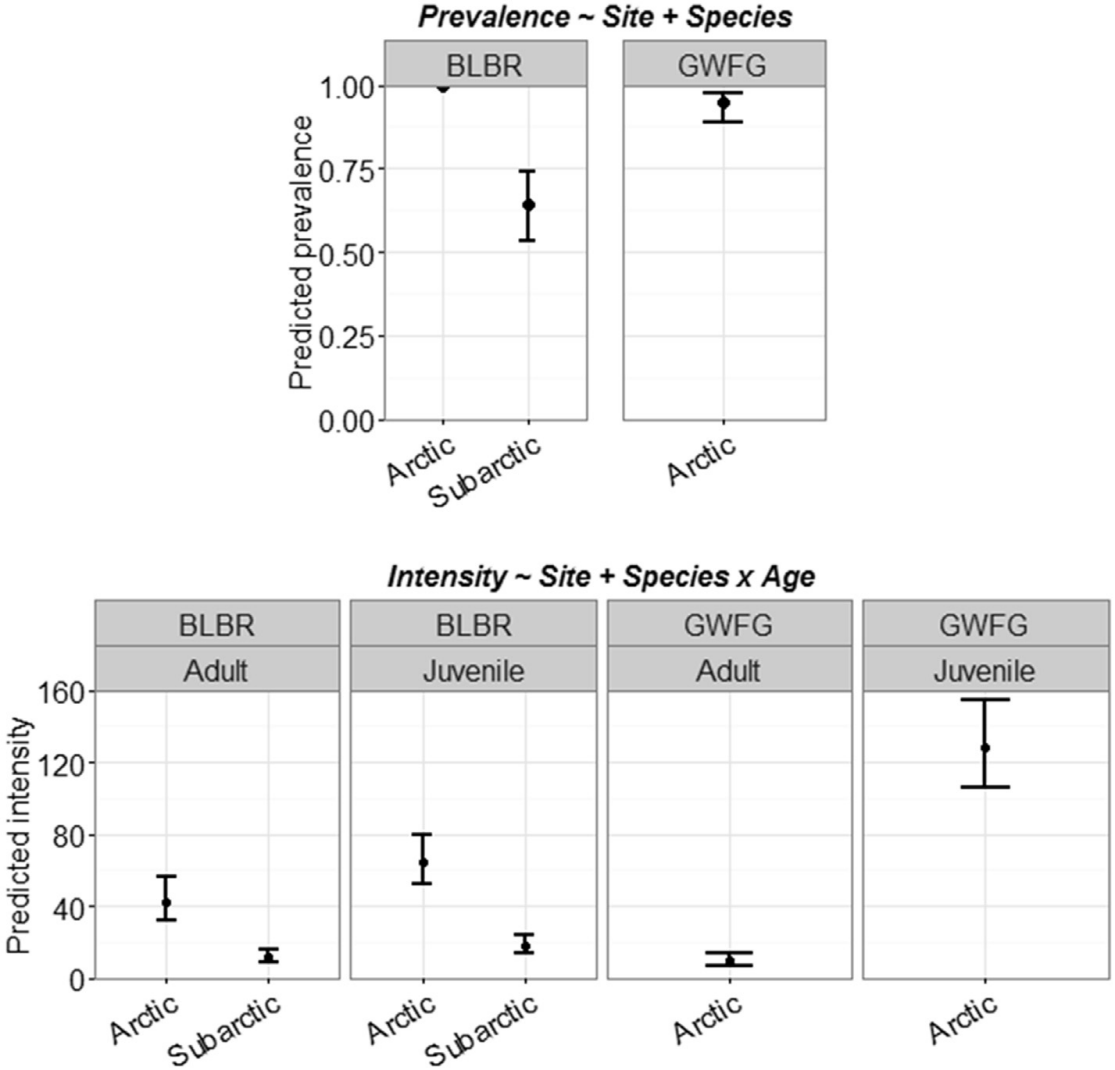
Predicted helminth infection intensity for combined cestodes identified in Pacific black brant (BLBR) and greater white-fronted geese (GWFG) collected in two locations (i.e., Arctic and Subarctic) in Alaska (2014). Circles represent predicted means and error bars denote 85% confidence intervals. Predictions are based off the most supported model from AIC_c_ selection and all interactions include lower-order effects (see title).

**Fig. 6:**
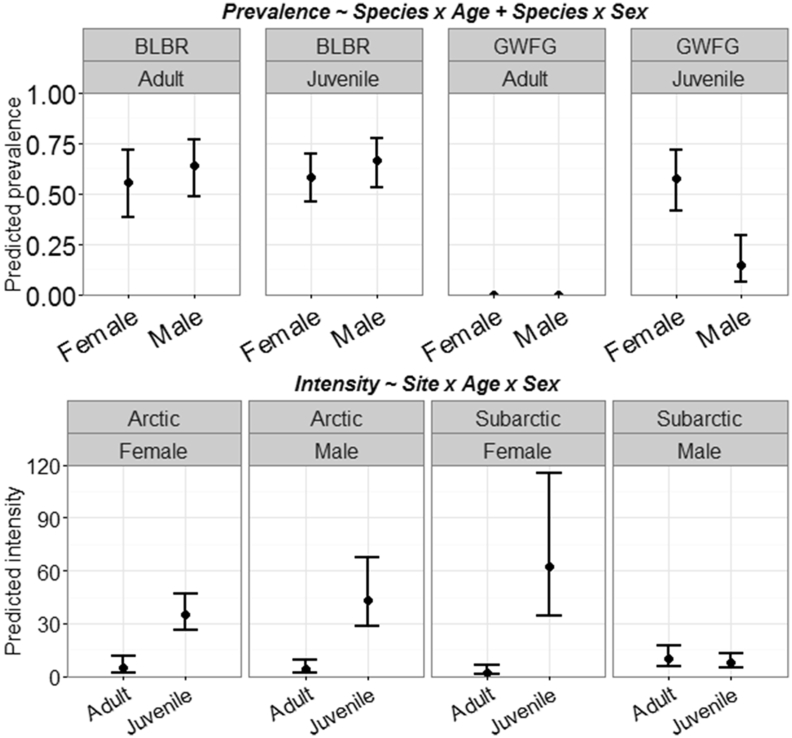
Predicted prevalence (top) and helminth infection intensity (bottom) for the cestode *Tschertkovilepis setigera* enumerated in Pacific black brant (BLBR) and greater white-fronted geese (GWFG) collected in Arctic and Subarctic Alaska (2014). Circles represent predicted means and error bars denote 85% confidence intervals. Predictions are based off the most supported model from AIC_c_ selection and all interactions include lower-order effects (see titles).

Individual cestode species generally had lower prevalence in Arctic geese (Table B2; Fig. B4). However, combined cestode intensity was similar between sexes, but higher in juveniles (108.09, 85% CI: 88.08–132.69) than adults (25.97, 19.49–34.63), Arctic (95.87, 85% CI: 77.98–117.95) than Subarctic geese (43.08, 85% CI: 33.47–55.46), and highest in juvenile white-fronted geese (141.90, 85% CI: 117.06–172.01; Fig. 5). Cestode prevalence and intensity varied by species; *T. setigera* had, on average, 32% (85% CI: 26–63%) higher prevalence and 65 (85% CI: 46–87) more worms per host in the Arctic, but this trend was driven primarily by juvenile white-fronted geese (Fig. 6). Conversely, *Wardoides nyrocae* had 28% (85% CI: 9–45%) higher prevalence and 33 (85% CI: 7–67) more worms per host in the Subarctic (Table B2; Figs. B4 and B6). *Drepanidotaenia lanceolata* was the only cestode that showed some support for sex-related infection intensity (Table 1); intensity was lower in Subarctic males (8.94, 85% CI: 5.45–14.68) than Subarctic females (31.81, 8% CI: 23.85–42.70) or Arctic birds (31.98, 85% CI: 22.78–45.37; Table B3 and Fig. 4).

### 3.2 Effects on body mass

After accounting for inherent differences in mass among species, sexes, and ages, an age by site interaction was supported where mass was lower for both age classes on the YKD, but the site effect was much greater for juveniles (Table B1). Further, the most supported model suggested mass decreased with combined trematode abundance (β = −1.94, 85% CI: −3.31, −0.59), which was driven by high trematode abundance in juvenile white-fronted geese. Brant harbored a maximum of 3 trematodes, whereas trematode abundance averaged 1.19 worms (range = 0–10) and 7.9 worms (range = 0–103) in adult and juvenile white-fronted geese, respectively.

### 3.3 Parasite correlations

Infection intensity between cestodes and nematodes was negatively correlated in Arctic geese (r = −0.28), but moderately positively correlated in Subarctic brant (r = 0.15; Table B6). Further, trematode and nematode intensity was generally negatively correlated (r = −0.11), except in Arctic brant (r = 0.53; Table B6). However, low trematode prevalence (0.07, n = 3) in Arctic brant make correlations unreliable. Cestodes and trematodes were moderately positively correlated in Subarctic brant (r = 0.18), but showed no relationship in other site or species groups (Table B6). Individual helminth species correlations varied widely among host species and locations (Table B7).

## 4 Discussion

Helminth community composition at high latitudes was similar between host species, sites, age classes, and sexes. However, helminth species richness, prevalence and intensity were structured within sampled populations consistent with helminth and host ecology. Further, all geese sampled were infected with at least one helminth species and all helminth species identified were present in 1) both age classes, providing evidence of transmission at high latitudes, 2) both species, suggesting broad host susceptibility, and 3) at both sites for all but one helminth species (i.e., *E. crami* was present in the Arctic only), suggesting conditions are suitable for transmission of a broad suite of helminths across a latitudinal gradient.

### 4.1 Geographic variation

We observed some variation in helminth infection characteristics by site; infection intensity of nematodes was higher in the Subarctic for the 3 individual nematode species evaluated, but this trend was not supported for total nematode intensity, and aggregation was similar for combined nematodes between sites. Helminth transmission dynamics are strongly influenced by weather (Stromberg, 1997) with individual species having optimal moisture and temperature ranges that maximize infectivity (Hudson et al., 1992). Therefore, responses to temperature differences are likely non-linear with positive responses to temperature up to a point at which further increases lower survival and infectivity (Molnár et al., 2013, Paull et al., 2012). However, studies suggest nematode abundance and infection intensity in definitive hosts increases with mean temperature assuming adequate moisture (Buscher, 1965, Hudson et al., 1992, Moss et al., 1993, Calvete and Estrada, 2003). Thus, infectivity may be higher in the warmer and wetter Subarctic. Further, higher host density (i.e., larger brant colonies) in the Subarctic may facilitate transmission of nematodes during their infective stage (Moss et al., 1993, Arneberg et al., 1998). Contrary to our predictions, infection intensity of combined cestodes was higher in the Arctic than Subarctic for brant, suggesting environmental conditions and availability of intermediate hosts did not limit cestode transmission. Further, helminth richness, prevalence rates, intensity, and community composition were similar in the Subarctic and Arctic despite differences in climate and habitat between the two areas.

For migratory waterfowl, geographically isolated populations may lead to seasonal variation in helminth diversity, prevalence, and species composition owed to differences in host behavior, habitat, diet, and environmental conditions (Buscher, 1965, Wallace and Pence, 1986, Altizer et al., 2006). Likewise, our results suggest several helminths identified likely infect birds exclusively on the breeding grounds in both the Arctic and Subarctic. Brant and white-fronted geese are allopatric outside of the breeding season, and movement between our two sites is limited to molt migrations of failed and non-breeding brant from the Subarctic to the Arctic in July–August. Therefore, the co-occurrence of the same species of parasites in both brant and white-fronted geese suggest either a) adults arrive at breeding areas infected with helminths and transmission continues at high latitudes, even among helminth species that require at least one intermediate host, or b) adults, like juveniles, become infected on the breeding grounds. Although brant helminth communities are unknown outside of breeding areas, Fedynich et al. (2005) surveyed helminths in wintering (Nov–Jan) white-fronted geese and identified 4 direct life cycle nematodes (*A. anseris, A. spatulatum, E. crami,* and *H. dispar*) that were also identified in this study suggesting nematodes have suitable environmental conditions to remain infective through most of the annual cycle. However, unlike nematodes, there was no overlap in white-fronted geese cestode or trematode communities between Fedynich et al. (2005) and our results suggesting little transmission between breeding and wintering grounds. *D. lanceolata* infections in snow geese declined through winter to become virtually non-existent by the end of spring migration (Forbes and Alisauskas, 1999). Concordantly, cestode prevalence (34%) and species richness (n = 2) were lower in white-fronted geese collected on the ACP in spring than observed in our study (Schiller, 1952). Therefore, cestodes and trematodes identified in our study likely persist on the breeding grounds either as dormant cysts or in resident intermediate hosts (McDonald, 1969, Forbes and Alisauskas, 1999).

Most helminths identified in our study commonly infect waterfowl, including geese during the breeding (Nerassen and Holmes, 1975, McLaughlin, 1990, Clinchy and Barker, 1994, Righi and Gauthier, 2002, Mellor and Rockwell, 2006) and non-breeding season (Tuggle and Crites, 1984, Purvis et al., 1997, Fedynich et al., 2005, Figuerola et al., 2005, Shutler et al., 2012). Further, species richness is similar to reports, though few, published of other high-latitude breeding geese (Schiller, 1954, Clinchy and Barker, 1994, Fedynich et al., 2005, Figuerola et al., 2005). In particular, cestodes identified here were also identified in geese that breed across Canada (McLaughlin and Burt, 1979, McLaughlin, 1990, Clinchy and Barker, 1994) and Europe or Western Russia (e.g., greylag geese; Figuerola et al., 2005). Despite this, we found prevalence of *T. Setigera*, and intensity of *T. setigera* and *D. lanceolata* were higher in Arctic than Subarctic sites, suggesting local conditions influence infection characteristics even in helminths with widespread distributions.

### 4.2 Age variation

Consistent with our predictions, structurally larger adults had higher infection intensities of nematodes, but lower cestode and trematode burdens. Thus, nematode intensity may be linked to exposure to contaminated feces from increased food intake among larger individuals in the population (Poulin, 1998) whereas juveniles likely consume more invertebrates than adults leading to higher cestode and trematode intensity (Sedinger, 1992).

### 4.3 Host species variation

In the Arctic, helminths requiring intermediate hosts were generally higher in white-fronted geese than brant; possibly because of differences in habitat use or diet between definitive hosts. Intermediate hosts in Arctic Alaska are likely environmentally segregated and more common in less-saline habitats frequented by white-fronted geese (e.g., freshwater inland lakes). However, nematode and cestode intensity were negatively correlated in our study. Similarly, Mellor and Rockwell (2006) found higher nematode intensities in snow goose goslings reared in coastal salt-marshes than inland freshwater habitats in Manitoba, Canada and suggested that fecal deposition by migrating and breeding birds along the coast may be the initial source of the parasites and regrazing by geese facilitates nematode transmission in these habitats. Thus, foraging on grazing lawns of salt-tolerant sedges may result in lower exposure to wetlands containing infected invertebrates, but greater contact with feces infected with nematodes, whereas foraging in shallow wetlands likely facilitates invertebrate consumption and dilutes fecal material.

We found weak and mixed patterns in sex-related helminth prevalence and intensity. Consistent with our predictions, trematode prevalence was higher in male white-fronted geese, but the only individual helminth to have a best supported prevalence model including sex (*T. tenuis*) showed the opposite trend; prevalence was higher in artic females and Subarctic males (Fig. 6). Further, infection intensity did not vary by combined helminth group, but one cestode (*D. lanceolata*) and one nematode (*T. tenuis*) had higher intensities in females. Sex-related variation in helminth infections may be a combination of morphology (e.g., the larger sex selecting larger or different food items with higher infection rates; Robinson et al., 2008, Skirnisson, 2015), immunocompetence (e.g., males have larger spleens and higher testosterone levels; Folstad and Karter, 1992), and life history characteristics (e.g., females increase food intake and subsequent exposure to helminths prior to egg laying; Skirnisson, 2015) and thus, patterns may not be clear among host and helminth species (Poulin, 1996).

We found trematode intensity was associated with lower body mass, but this trend was driven by juvenile white-fronted geese, which was the only group that suffered intense infections of trematodes. Pre-fledging waterfowl are highly susceptible to parasitic infection due to naïve immune systems and helminth infections have been shown to negatively affect survival of young waterfowl (Wehr and Herman, 1954, Graczyk and Shiff, 1993). However, on the ACP, white-fronted gosling growth rates are high relative to other breeding areas (T. Fondell and B. Meixell, U.S. Geological Survey, unpublished data) suggesting external stressors (e.g., food limitation, contaminants) are not negatively affecting goslings.

A pattern of warming and increased season length has been apparent in the Arctic for many years (Marshall et al., 2014). However, a lack of detailed historic data and spatial and temporal heterogeneity in infection characteristics, intermediate and definitive host populations, and environmental conditions make it difficult to establish a baseline and subsequently determine if or how parasite communities are responding to these changes. Moreover, helminth prevalence and infection intensity likely vary year-to-year as a function of temperature, precipitation, and host density and distribution. Thus, a comprehensive examination of helminth community structure through broad geographic areas across multiple years is necessary to untangle the complex interactions between parasites and hosts at high latitudes and fully elucidate current states in order to evaluate change into the future (Hoberg et al., 2008, Hoberg et al., 2013). Recently, methods have emerged that may be useful tools for predicting parasite and host responses to continued warming at high latitudes. For example, Hoberg et al. (2008) outlined a process to detect climate-related changes in parasite emergence and host consequences that integrated data from field studies, laboratory experiments, and archived specimens. Newly developed multi-trophic models of parasite-host communities are able to link habitat, environmental conditions, and host-parasite tolerances to predict range shifts of helminths under climate change scenarios (Morgan et al., 2007, Pickles et al., 2013). Additionally, Molnár et al. (2013) described a metabolic model that provides a mechanistic understanding of how environmental changes may affect parasite fitness. These advances hold promise for improving our understanding of host-parasite relationships at high latitudes under continued climate change. Accordingly, our results can be used to populate more complex models and are a first step toward understanding transmission dynamics at high latitudes.

## Figures and Tables

**Table 1:** The number of individuals infected, apparent prevalence, apparent infection intensity, and Boulinier's *J* aggregation index values for 13 individual helminth species and combined helminth guilds detected in Pacific black brant and greater white-fronted geese collected in Arctic and Subarctic Alaska (2014). We provide 85% confidence intervals derived from the Sterne (1954) method (prevalence) or bootstrapping (intensity and J). We present the most supported model (based on AICc model selection, see Methods) from an evaluation of age (juvenile or adult), species (brant or white-fronted geese), sex, and site (Arctic or Subarctic) effects on prevalence and intensity. Models with interactive effects also include main effects. We only evaluated relationships for parasites species and classes that had at least 10 individuals infected, and limited models to main effects only if < 40 birds were infected. Note: Sample sizes can be found in Table B5.

Helminth	# Infected (n = 141)	Prevalence	Intensity	*J*	Prevalence Model	Intensity Model
Nematodes	136	0.96 (0.94–0.99)	23.51 (20.82–26.77)	1.02 (0.8–1.48)	–	∼ Age
Cestodes	131	0.93 (0.9–0.96)	81.78 (72.15–95.72)	1.44 (1.1–2.03)	–	∼ Site + Species × Age
Trematodes	20	0.14 (0.1–0.18)	18.2 (10.98–32.95)	25.8 (13.91–56.81)	∼Species	∼ Species + Age

*T. tenuis*	119	0.84 (0.8–0.89)	11.31 (9.87–13.55)	1.71 (1.28–2.54)	∼Sex × Site	∼ Site + Sex + Species × Age
*H. dispar*	90	0.64 (0.58–0.7)	12.59 (10.09–16.22)	3.94 (2.76–6.81)	∼Species + Age	∼ Site + Sex
*A. anseris*	36	0.26 (0.2–0.31)	11.92 (8.76–15.89)	8.73 (5.28–15.12)	∼Species + Site	∼ Age + Site
*A. spatulatum*	7	0.05 (0.03–0.08)	1.86 (1.29–2.14)	14.77	–	–
*E. crami*	6	0.04 (0.02–0.07)	11.33 (5.83–22.17)	48.26	–	–
*T. striata*	17	0.12 (0.08–0.16)	12.24 (3.88–29.77)	68.67 (33.61–133.86)	–	–
*T. setigera*	123	0.87 (0.83–0.91)	66.51 (56.58–80.83)	2.13 (1.59–3.07)	∼Species + Site	∼ Site + Species × Age
*D. lanceolata*	66	0.47 (0.41–0.53)	27.02 (21.26–37.7)	6.94 (4.71–12.09)	∼Species × Age + Species x Sex	∼ Age × Sex × Site
*W. nyrocae*	26	0.18 (0.14–0.23)	28.81 (20.46–39.77)	12.44 (7.58–24.15)	∼Age + Site	∼ Age + Site
*N. attenuatus*	16	0.11 (0.08–0.15)	20.19 (11.16–36.63)	29.32 (15.64–70.34)	–	–
U. trematode	5	0.04 (0.01–0.06)	8.2 (1–14.4)	89.1	–	–
