# Supplementary material for: Helminth community structure in two species of arctic-breeding waterfowl
Source: Int J Parasitol Parasites Wildl. 2016 Sep 17;5(3):263–72. doi: 10.1016/j.ijppaw.2016.09.002 (PMC5040642; doi:10.1016/j.ijppaw.2016.09.002)
Supplement: Supplementary file 1 [file mmc1.docx]

**Supplementary Material A: Specimen Fate**

Voucher specimens are deposited in the Sam Houston State University Natural History Parasite Collection (SHSUP), Huntsville, Texas 77341. The voucher series consists of specimens deposited in the following lots:

*Amidostomum anseris—*41 vials: SHSUP 000,494-000,495; 000,497-000,505; 000,507; 000,536; 000,587; 000,589-000,590; 000,601; 000,626-000,629; 000,637; 000,642; 000,644; 000,648-000,649; 000,653; 001,009-001,012; 001,014; 001,038; 001,041;001,054-001,055; 001,143-001,147.

*Amidostomum spatulatum—*3 vials: SHSUP 000,496; 000,506; 000,638.

*Drepanidotaenia lanceolata*—152 vials: SHSUP 000,602-000,606; 000,654-000,070; 000,810-000,831; 000,955-000,965; 001,021-001,032; 001,076-001,080; 001,141-001,157; 001,161; 001,169; 001,174; 001,183; 001,185; 001,187; 001,192; 001,197; 001,209-001,211; 001,213-001,214; 001,222; 001,228-001,230; 001,323-001,360.

*Epomidiostomum crami*—5 vials: SHSUP 000,630-000,634

*Heterakis dispar*—95 vials: SHSUP 000,508-000,525; 000,527; 000,542-000,547; 000,607-000,614; 000,641; 000,647; 000,652; 000,988-000,996; 001,015-001,016; 001,035; 001,039; 001,052-001,053; 001,056-001,057; 001,070; 001,072; 001,092; 001,119-001,122; 001,163; 001,173; 001,178; 001,190; 001,195; 001,202-001,203; 001,234; 001,379-001,405.

*Notocotylus attenuatus*—41 vials: SHSUP 000,484-000,493; 000,578-000,585; 000,593-000,595; 000,599; 001,102-001,116; 001,165; 001,478-001,480.

*Tetrameres striata*—17 vials: SHSUP 000,540-000,541; 000,592; 000,635; 000,643; 000,645; 001,090-001,091; 001,123-001,124; 001,227; 001,448-001,453.

*Trichostrongylus tenuis*—119 vials: SHSUP 000,548-000,577; 000,586; 000,588; 000,591; 000,600; 000,615-000,625; 000,636; 000,639-000,640; 000,646; 000,650-000,651; 000,997-001,000; 001,001-001,008; 001,017-001,018; 001,051; 001,071; 001,093-001,094; 001,117-001,118; 001,162; 001,164; 001,172; 001,177; 001,181; 001,189; 001,194; 001,196; 001,201; 001,204; 001,226; 001,406-001,442.

*Tschertkovilepis setigera­*—409 vials: SHSUP 000,671-000,801; 000,806-000,808; 000,832-000,908; 000,966-000,987; 001,019-001,020; 001,036-001,037; 001,040; 001,042-001,050; 001,075; 001,081-001,088; 001,125-001,140; 001,160; 001,166-001,168; 001,170-001,171; 001,175-001,176; 001,179-001,180; 001,184; 001,186; 001,188; 001,191;001,193; 001,198-001,200; 001,205-001,208; 001,212; 001,224-001,225; 001,231-001,233; 001,235-001,322;001,361-001,378.

*Wardoides nyrocae*—60 vials: SHSUP 000,809; 000,909-000,954; 001,033-001,034; 001,058-001,060; 001,159; 001,074; 001,089; 001,158; 001,215; 001,223; 001,454-001,455.

**Supplementary Material B:**

**Table B1.** Summary of mass of Pacific black brant (BLBR) and greater white-fronted geese (GWFG) collected in Arctic and Subarctic Alaska, July-August 2014. We present mean mass with standard deviation (SD) and 85% confidence intervals.

| Species | Site | Age | Sex | Mass (g) | SD | 85% LCI | 85% UCI |
| --- | --- | --- | --- | --- | --- | --- | --- |
| BLBR | Arctic | Adult | Female | 1277.6 | 112.7 | 1115.3 | 1439.9 |
|  |  |  | Male | 1494.9 | 85.3 | 1372.0 | 1617.7 |
|  |  | Juvenile | Female | 916.3 | 111.8 | 755.4 | 1077.3 |
|  |  |  | Male | 921.9 | 162.7 | 687.6 | 1156.2 |
|  | Subarctic | Adult | Female | 1075.2 | 78.9 | 961.6 | 1188.8 |
|  |  |  | Male | 1276.6 | 52.9 | 1200.5 | 1352.7 |
|  |  | Juvenile | Female | 528.4 | 181.9 | 266.5 | 790.3 |
|  |  |  | Male | 611.3 | 169.1 | 367.7 | 854.8 |
| GWFG | Arctic | Adult | Female | 2183.4 | 90.5 | 2053.0 | 2313.7 |
|  |  |  | Male | 2535.8 | 87.5 | 2409.8 | 2661.7 |
|  |  | Juvenile | Female | 1324.2 | 169.7 | 1079.9 | 1568.5 |
|  |  |  | Male | 1497.6 | 180.8 | 1237.2 | 1758.0 |

**Table B2.** Apparent mean helminth prevalence (i.e., proportion infected) of adult and juvenile Pacific black brant (BLBR) and greater white-fronted geese (GWFG) of both sexes collected in Arctic and Subarctic Alaska, 2014. We report 85% confidence intervals (CIs) in parentheses and did not estimate CIs for mean intensities with < 3 birds infected. We derived CIs using the Sterne (1954) method.

|  | | **BLBR** | | | | | | | | | | | **GWFG** | | | | |
| --- | --- | --- | --- | --- | --- | --- | --- | --- | --- | --- | --- | --- | --- | --- | --- | --- | --- |
| **PREVALENCE** | | **Subarctic** | | | | | **Arctic** | | | | | | | | | | |
|  | | **Adult** | | **Juvenile** | | | **Adult** | | | **Juvenile** | | | **Adult** | | **Juvenile** | | |
| **Helminth** | **Male** | | **Female** | | **Male** | **Female** | | **Male** | **Female** | | **Male** | **Female** | **Male** | **Female** | | **Male** | **Female** |
| Nematodes | 1 (0.83–1) | | 1 (0.69–1) | | 1 (0.89–1) | 1 (0.84–1) | | 1 (0.69–1) | 1 (0.79–1) | | 1 (0.81–1) | 0.95 (0.81–1) | 1 (0.77–1) | 1 (0.79–1) | | 0.82 (0.63–0.96) | 1 (0.85–1) |
| Cestodes | 1 (0.83–1) | | 1 (0.69–1) | | 0.81 (0.61–0.96) | 0.64 (0.36–0.88) | | 1 (0.69–1) | 1 (0.79–1) | | 1 (0.81–1) | 1 (0.91–1) | 0.86 (0.56–1) | 0.88 (0.6–1) | | 0.94 (0.79–1) | 1 (0.85–1) |
| Trematodes | 0.1 (0–0.33) | | 0 (0–0.31) | | 0.19 (0.04–0.39) | 0 (0–0.16) | | 0 (0–0.31) | 0.13 (0–0.4) | | 0 (0–0.19) | 0.11 (0.01–0.27) | 0.43 (0.13–0.75) | 0.13 (0–0.4) | | 0.24 (0.07–0.45) | 0.17 (0.02–0.4) |
|  |  | |  | |  |  | |  |  | |  |  |  |  | |  |  |
| *T. tenuis* | 0.8 (0.54–0.98) | | 0.8 (0.44–1) | | 0.75 (0.53–0.92) | 0.64 (0.36–0.88) | | 1 (0.69–1) | 1 (0.79–1) | | 0.78 (0.5–0.97) | 0.95 (0.81–1) | 0.71 (0.39–0.96) | 1 (0.79–1) | | 0.76 (0.55–0.93) | 0.92 (0.72–1) |
| *H. dispar* | 0.6 (0.32–0.86) | | 0.6 (0.23–0.92) | | 0.81 (0.61–0.96) | 1 (0.84–1) | | 0.6 (0.23–0.92) | 0.88 (0.6–1) | | 1 (0.81–1) | 0.95 (0.81–1) | 0.29 (0.04–0.61) | 0.25 (0.03–0.55) | | 0.35 (0.15–0.58) | 0.33 (0.11–0.59) |
| *A. anseris* | 0 (0–0.17) | | 0.2 (0–0.56) | | 0.44 (0.22–0.67) | 0.55 (0.27–0.8) | | 1 (0–0.31) | 0 (0–0.21) | | 0.11 (0–0.36) | 0.05 (0–0.19) | 0.86 (0.56–1) | 0.88 (0.6–1) | | 0.24 (0.07–0.45) | 0.25 (0.06–0.5) |
| *A. spatulatum* | 0 (0–0.17) | | 0 (0–0.31) | | 0.06 (0–0.22) | 0 (0–0.16) | | 0.2 (0–0.56) | 0 (0–0.21) | | 0 (0–0.19) | 0 (0–0.09) | 0.14 (0–0.44) | 0.25 (0.03–0.55) | | 0.06 (0–0.21) | 0.08 (0–0.28) |
| *E. crami* | 0 (0–0.17) | | 0 (0–0.31) | | 0 (0–0.11) | 0 (0–0.16) | | 0 (0–0.31) | 0 (0–0.21) | | 0.11 (0–0.36) | 0 (0–0.09) | 0.29 (0.04–0.61) | 0.38 (0.1–0.69) | | 0 (0–0.11) | 0 (0–0.15) |
| *T. striata* | 0.2 (0.02–0.46) | | 0.2 (0–0.56) | | 0 (0–0.11) | 0 (0–0.16) | | 0.4 (0.08–0.77) | 0.13 (0–0.4) | | 0.11 (0–0.36) | 0.21 (0.06–0.4) | 0.29 (0.04–0.61) | 0.38 (0.1–0.69) | | 0.06 (0–0.21) | 0 (0–0.15) |
| *T. setigera* | 0.8 (0.54–0.98) | | 0.6 (0.23–0.92) | | 0.69 (0.46–0.88) | 0.45 (0.2–0.73) | | 1 (0.69–1) | 1 (0.79–1) | | 1 (0.81–1) | 1 (0.91–1) | 0.86 (0.56–1) | 0.88 (0.6–1) | | 0.94 (0.79–1) | 1 (0.85–1) |
| *D. lanceolata* | 0.7 (0.42–0.93) | | 0.6 (0.23–0.92) | | 0.63 (0.39–0.83) | 0.45 (0.2–0.73) | | 0.6 (0.23–0.92) | 0.5 (0.2–0.8) | | 0.78 (0.5–0.97) | 0.63 (0.42–0.83) | 0 (0–0.23) | 0 (0–0.21) | | 0.06 (0–0.21) | 0.33 (0.11–0.59) |
| *W. nyrocae* | 0.5 (0.22–0.78) | | 0.6 (0.23–0.92) | | 0.31 (0.12–0.54) | 0.27 (0.07–0.54) | | 0.2 (0–0.56) | 0.13 (0–0.4) | | 0.22 (0.03–0.5) | 0.05 (0–0.19) | 0.14 (0–0.44) | 0.13 (0–0.4) | | 0.06 (0–0.21) | 0 (0–0.15) |
| *N. attenuatus* | 0 (0–0.17) | | 0 (0–0.31) | | 0.19 (0.04–0.39) | 0 (0–0.16) | | 0 (0–0.31) | 0.13 (0–0.4) | | 0 (0–0.19) | 0.11 (0.01–0.27) | 0.14 (0–0.44) | 0.13 (0–0.4) | | 0.24 (0.07–0.45) | 0.17 (0.02–0.4) |
| U. trematode | 0.1 (0–0.33) | | 0 (0–0.31) | | 0 (0–0.11) | 0 (0–0.16) | | 0 (0–0.31) | 0 (0–0.21) | | 0 (0–0.19) | 0 (0–0.09) | 0.29 (0.04–0.61) | 0 (0–0.21) | | 0.06 (0–0.21) | 0 (0–0.15) |

**Table B3.** Apparent mean helminth infection intensity (i.e., the number of individual worms in infected individuals) of adult and juvenile Pacific black brant (BLBR) and greater white-fronted geese (GWFG) of both sexes collected in Arctic and Subarctic Alaska, 2014. We report 85% confidence intervals (CIs) in parentheses and did not estimate CIs for mean intensities with < 3 birds infected. We derived CIs using the Sterne (1954) method.

|  | | **BLBR** | | | | | | | | | | **GWFG** | | | | |
| --- | --- | --- | --- | --- | --- | --- | --- | --- | --- | --- | --- | --- | --- | --- | --- | --- |
| **INTENSITY** | | **Subarctic** | | | | | **Arctic** | | | | | | | | | |
|  | | **Adult** | | **Juvenile** | | | **Adult** | | | **Juvenile** | | **Adult** | | **Juvenile** | | |
| **Helminth** | **Male** | | **Female** | | **Male** | **Female** | | **Male** | **Female** | **Male** | **Female** | **Male** | **Female** | | **Male** | **Female** |
| Nematodes | 29 (19.86–28.24) | | 28 (19.93–27.97) | | 25.94 (20.43–28.68) | 18.18 (20.32–27.99) | | 19.2 (20.05–27.75) | 34.5 (20.34–28.2) | 22.56 (20.09–28.98) | 23.94 (20.28–28.24) | 26.14 (13.71–33.29) | 37 (24.63–59.36) | | 16.5 (5.36–56.17) | 10.5 (5.92–16.23) |
| Cestodes | 33.1 (67.74–100.44) | | 10.8 (68.53–98.66) | | 43.77 (68.39–99.91) | 78.71 (69.02–103.6) | | 55.6 (67.83–100.63) | 35.13 (68.39–100.24) | 113.44 (68.71–100.89) | 87.63 (67.69–99.45) | 10.83 (5–23.67) | 11.14 (7.12–15.29) | | 166.44 (114.64–272.21) | 147.42 (95.99–198.4) |
| Trematodes | 1 | | 0 (0–0) | | 1.33 (8–36.12) | 0 | | 0 | 3 | 0 | 2.5 | 3 (1–4.67) | 10 | | 46.25 (8.25–81.75) | 70.5 |
|  |  | |  | |  |  | |  |  |  |  |  |  | |  |  |
| *T. tenuis* | 9.88 (9.13–13.82) | | 20.75 (9.29–14.13) | | 4 (9.17–14.2) | 10 (9.24–14.25) | | 15.4 (9.2–14.03) | 21.13 (9.19–14.09) | 14.14 (9.23–14.06) | 13.44 (9.28–14.21) | 5.4 (2.4–10) | 9.25 (4.75–14.38) | | 4.77 (2.69–9.16) | 8.64 (4.64–15.32) |
| *H. dispar* | 33.17 (9.9–18.05) | | 18 (9.51–17.82) | | 20.46 (9.38–17.67) | 7.55 (9.71–17.95) | | 2 (9.54–17.99) | 14.71 (9.62–18.22) | 10.56 (9.59–18.29) | 10 (9.76–17.96) | 6 (0–0) | 3 | | 3 (1.17–7) | 5.5 (2.25–8.25) |
| *A. anseris* | 0 | | 1 | | 14 (7.57–17.42) | 7.83 (8.11–18.87) | | 0 | 0 | 2 | 2 | 14.5 (6.33–21.33) | 25.57 (15–46.33) | | 1.25 (1–1.5) | 2.67 (2–3.33) |
| *A. spatulatum* | 0 | | 0 | | 3 | 0 | | 2 | 0 | 0 | 0 | 3 | 1.5 | | 1 | 1 |
| *E. crami* | 0 | | 0 | | 0 | 0 | | 0 | 0 | 1 | 0 | 23.5 | 6.67 (3–9.33) | | 0 | 0 |
| *T. striata* | 6 (3.53–38) | | 2 | | 0 | 0 | | 5.5 | 4 | 6 | 1.75 (3.59–44.47) | 3.5 | 4.67 (1–7.67) | | 145 | 0 |
| *T. setigera* | 13.13 (54.02–87.63) | | 11.33 (54.42–87.3) | | 20.27 (53.39–86.22) | 11 (53.57–86.86) | | 52.4 (53.33–85.17) | 32.75 (53.2–86.25) | 75.89 (54.12–84.83) | 60.89 (54.03–84.34) | 10.67 (4.83–23.55) | 10.71 (7.29–14.57) | | 163 (113.63–282.56) | 146.25 (98.77–195.93) |
| *D. lanceolata* | 10.14 (18.95–42.68) | | 2.33 (18.62–42.01) | | 8.1 (19.08–43.31) | 62.6 (19.4–41.41) | | 5 (19.11–41) | 4.25 (19.71–43.06) | 43.86 (19.15–43.83) | 41.92 (18.91–43.57) | 0 | 0 | | 48 | 3.5 (1.5–5) |
| *W. nyrocae* | 31 (16.21–45.04) | | 4.33 (16.54–45.55) | | 53 (17.35–44.38) | 61 (17.23–45.71) | | 1 | 2 | 15.5 | 5 | 1 | 3 | | 7 | 0 |
| *N. attenuatus* | 0 | | 0 | | 1.33 (8.06–42.08) | 0 | | 0 | 3 | 0 | 2.5 | 2 | 10 | | 38.25 (10.5–84) | 70.5 |
| U. trematode | 1 | | 0 | | 0 | 0 | | 0 | 0 | 0 | 0 | 3.5 | 0 | | 32 | 0 |

**Table B4:** Number of greater white fronted geese (GWFG) and Pacific black brant (BLBR) collected (*n*) and infected with helminth guilds and individual species in Subarctic and Arctic Alaska, 2014.

|  |  | **BLBR** | | | | | | | | **GWFG** | | | |
| --- | --- | --- | --- | --- | --- | --- | --- | --- | --- | --- | --- | --- | --- |
|  |  | **Subarctic** | | | | **Arctic** | | | | | | | |
|  |  | **Adult** | | **Juvenile** | | **Adult** | | **Juvenile** | | **Adult** | | **Juvenile** | |
|  | **Overall** | **Male** | **Female** | **Male** | **Female** | **Male** | **Female** | **Male** | **Female** | **Male** | **Female** | **Male** | **Female** |
| ***n*** | 141 | 10 | 5 | 16 | 11 | 8 | 5 | 9 | 19 | 8 | 8 | 21 | 21 |
| **Helminth Group** |  |  |  |  |  |  |  |  |  |  |  |  |  |
| Nematodes | 136 | 10 | 5 | 16 | 11 | 5 | 8 | 9 | 18 | 7 | 8 | 14 | 12 |
| Cestodes | 131 | 10 | 5 | 13 | 7 | 5 | 8 | 9 | 19 | 6 | 7 | 16 | 12 |
| Trematodes | 20 | 1 | 0 | 3 | 0 | 0 | 1 | 0 | 2 | 3 | 1 | 4 | 2 |
| **Helminth Species** |  |  |  |  |  |  |  |  |  |  |  |  |  |
| *T. tenuis* | 119 | 8 | 4 | 12 | 7 | 5 | 8 | 7 | 18 | 5 | 8 | 13 | 11 |
| *H. dispar* | 90 | 6 | 3 | 13 | 11 | 3 | 7 | 9 | 18 | 2 | 2 | 6 | 4 |
| *A. anseris* | 36 | 0 | 1 | 7 | 6 | 0 | 0 | 1 | 1 | 6 | 7 | 4 | 3 |
| *A. spatulatum* | 7 | 0 | 0 | 1 | 0 | 1 | 0 | 0 | 0 | 1 | 2 | 1 | 1 |
| *E. crami* | 6 | 0 | 0 | 0 | 0 | 0 | 0 | 1 | 0 | 2 | 3 | 0 | 0 |
| *T. striata* | 17 | 2 | 1 | 0 | 0 | 2 | 1 | 1 | 4 | 2 | 3 | 1 | 0 |
| *T. setigera* | 123 | 8 | 3 | 11 | 5 | 5 | 8 | 9 | 19 | 6 | 7 | 16 | 12 |
| *D. lanceolata* | 66 | 7 | 3 | 10 | 5 | 3 | 4 | 7 | 12 | 0 | 0 | 1 | 4 |
| *W. nyrocae* | 26 | 5 | 3 | 5 | 3 | 1 | 1 | 2 | 1 | 1 | 1 | 1 | 0 |
| *N. attenuatus* | 16 | 0 | 0 | 3 | 0 | 0 | 1 | 0 | 2 | 1 | 1 | 4 | 2 |
| Unk. trematode | 5 | 1 | 0 | 0 | 0 | 0 | 0 | 0 | 0 | 2 | 0 | 1 | 0 |

**Table B5.** The number of individuals infected, aggregation of parasites (Boulinier’s J), and associated 85% or 95% confidence intervals for 13 helminth species and 3 helminth guilds detected in Pacific black brant (BLBR) and greater white-fronted geese (GWFG) collected in two areas in Alaska, 2014. Estimates are broken down into age classes, sexes, host species, and sites. We restricted CI estimates for Boulinier’s *J* to groups with > 10 individuals infected because preliminary analyses suggested this was the minimum necessary to accurately simulate *J* and derive bootstrapped confidence intervals (Sherrard-Smith et al. 2015).

| **Group** | **# infected** | **Helminth** | ***J*** | **LCI85** | **UCI85** | **LCI95** | **UCI95** |
| --- | --- | --- | --- | --- | --- | --- | --- |
| Overall  (n = 141) | 119 | *Trichostrongylus tenuis* | 1.707867 | 1.281882 | 2.541198 | 1.151466 | 2.541198 |
|  | 90 | *Heterakis dispar* | 3.938071 | 2.75931 | 6.854551 | 2.472754 | 6.854551 |
|  | 36 | *Amidostomum anseris* | 8.727586 | 5.281473 | 15.11614 | 4.600363 | 15.11614 |
|  | 7 | *Amidostomum spatulatum* | 14.76606 |  |  |  |  |
|  | 6 | *Epomidiostomum crami* | 48.25674 |  |  |  |  |
|  | 17 | *Tetrameres striata* | 68.66834 | 33.61019 | 133.859 | 25.51675 | 133.859 |
|  | 123 | *Tschertkovilepis setigera* | 2.131341 | 1.593928 | 3.072103 | 1.432127 | 3.072103 |
|  | 66 | *Drepanidotaenia lanceolata* | 6.938892 | 4.707273 | 12.08576 | 4.184179 | 12.08576 |
|  | 26 | *Wardoides nyrocae* | 12.43651 | 7.583349 | 24.14513 | 6.810116 | 24.14513 |
|  | 16 | *Notocotylus attenuatus* | 29.31726 | 15.64458 | 70.33633 | 13.33533 | 70.33633 |
|  | 5 | U. trematode | 89.10369 |  |  |  |  |
|  | 136 | Nematodes | 1.019165 | 0.799165 | 1.475144 | 0.745275 | 1.475144 |
|  | 131 | Cestodes | 1.440008 | 1.099335 | 2.033369 | 1.022343 | 2.033369 |
|  | 20 | Trematodes | 25.79812 | 13.91265 | 56.8108 | 12.08766 | 56.8108 |
| Juvenile  (n = 97) | 81 | *Trichostrongylus tenuis* | 2.124136 | 1.485409 | 3.397309 | 1.341907 | 3.397309 |
|  | 67 | *Heterakis dispar* | 2.419892 | 1.695223 | 3.843665 | 1.504365 | 3.843665 |
|  | 22 | *Amidostomum anseris* | 14.55193 | 8.032232 | 33.55575 | 6.788608 | 33.55575 |
|  | 0 | *Amidostomum spatulatum* |  |  |  |  |  |
|  | 0 | *Epomidiostomum crami* |  |  |  |  |  |
|  | 6 | *Tetrameres striata* | 81.33122 |  |  |  |  |
|  | 85 | *Tschertkovilepis setigera* | 1.573616 | 1.150825 | 2.283816 | 1.044582 | 2.283816 |
|  | 49 | *Drepanidotaenia lanceolata* | 5.170165 | 3.309859 | 9.358696 | 3.014249 | 9.358696 |
|  | 14 | *Wardoides nyrocae* | 11.99955 | 6.926106 | 24.60632 | 5.944744 | 24.60632 |
|  | 13 | *Notocotylus attenuatus* | 21.84334 | 11.15703 | 43.23527 | 9.389097 | 43.23527 |
|  | 2 | U. trematode | 93.02934 |  |  |  |  |
|  | 93 | Nematodes | 1.18422 | 0.868637 | 1.784766 | 0.790247 | 1.784766 |
|  | 89 | Cestodes | 1.016215 | 0.756212 | 1.527261 | 0.697052 | 1.527261 |
|  | 14 | Trematodes | 19.90657 | 10.54776 | 42.43823 | 9.050517 | 42.43823 |
| Adult  (n = 44) | 38 | *Trichostrongylus tenuis* | 1.085475 | 0.676109 | 1.795367 | 0.602092 | 1.795367 |
|  | 23 | *Heterakis dispar* | 6.639195 | 3.426035 | 15.54678 | 2.872951 | 15.54678 |
|  | 14 | *Amidostomum anseris* | 4.2742 | 2.363388 | 9.525039 | 2.033838 | 9.525039 |
|  | 4 | *Amidostomum spatulatum* | 8.132075 |  |  |  |  |
|  | 5 | *Epomidiostomum crami* | 14.83648 |  |  |  |  |
|  | 11 | *Tetrameres striata* | 3.581164 | 1.664383 | 8.245981 | 1.308925 | 8.245981 |
|  | 38 | *Tschertkovilepis setigera* | 1.900415 | 1.191532 | 3.548084 | 1.020198 | 3.548084 |
|  | 17 | *Drepanidotaenia lanceolata* | 2.584431 | 1.418752 | 5.24996 | 1.143594 | 5.24996 |
|  | 12 | *Wardoides nyrocae* | 12.53758 | 6.266575 | 27.68441 | 5.155863 | 27.68441 |
|  | 3 | *Notocotylus attenuatus* | 21.07423 |  |  |  |  |
|  | 3 | U. trematode | 26.39623 |  |  |  |  |
|  | 43 | Nematodes | 0.710557 | 0.461743 | 1.196593 | 0.410534 | 1.196593 |
|  | 42 | Cestodes | 1.263471 | 0.795296 | 2.180417 | 0.683574 | 2.180417 |
|  | 6 | Trematodes | 10.64785 |  |  |  |  |
| BLBR  (n = 83) | 69 | *Trichostrongylus tenuis* | 1.782731 | 1.261384 | 2.868048 | 1.150488 | 2.868048 |
|  | 70 | *Heterakis dispar* | 2.537336 | 1.698954 | 4.270129 | 1.527961 | 4.270129 |
|  | 0 | *Amidostomum anseris* |  |  |  |  |  |
|  | 0 | *Amidostomum spatulatum* |  |  |  |  |  |
|  | 0 | *Epomidiostomum crami* |  |  |  |  |  |
|  | 11 | *Tetrameres striata* | 7.7914 | 3.62894 | 19.04351 | 2.770237 | 19.04351 |
|  | 68 | *Tschertkovilepis setigera* | 1.606363 | 1.135543 | 2.584603 | 1.015439 | 2.584603 |
|  | 51 | *Drepanidotaenia lanceolata* | 5.155538 | 3.226978 | 9.679189 | 2.812057 | 9.679189 |
|  | 21 | *Wardoides nyrocae* | 8.470459 | 4.798568 | 16.5134 | 4.10677 | 16.5134 |
|  | 6 | *Notocotylus attenuatus* | 10.05335 |  |  |  |  |
|  | 0 | U. trematode |  |  |  |  |  |
|  | 82 | Nematodes | 0.734685 | 0.543508 | 1.058795 | 0.483898 | 1.058795 |
|  | 76 | Cestodes | 1.338421 | 0.9393 | 2.100308 | 0.85589 | 2.100308 |
|  | 7 | Trematodes | 8.276553 |  |  |  |  |
| GWFG  (n = 58) | 50 | *Trichostrongylus tenuis* | 1.436347 | 0.93714 | 2.407266 | 0.835333 | 2.407266 |
|  | 20 | *Heterakis dispar* | 8.598859 | 4.48783 | 21.15264 | 3.648213 | 21.15264 |
|  | 20 | *Amidostomum anseris* | 5.378755 | 2.976006 | 10.68565 | 2.615379 | 10.68565 |
|  | 5 | *Amidostomum spatulatum* | 8.638968 |  |  |  |  |
|  | 5 | *Epomidiostomum crami* | 19.87713 |  |  |  |  |
|  | 6 | *Tetrameres striata* | 43.72245 |  |  |  |  |
|  | 55 | *Tschertkovilepis setigera* | 1.473884 | 0.987198 | 2.552971 | 0.879025 | 2.552971 |
|  | 15 | *Drepanidotaenia lanceolata* | 12.48904 | 6.381009 | 28.11744 | 5.407685 | 28.11744 |
|  | 5 | *Wardoides nyrocae* | 29.26861 |  |  |  |  |
|  | 10 | *Notocotylus attenuatus* | 12.44501 | 6.265064 | 28.54798 | 5.386715 | 28.54798 |
|  | 4 | U. trematode | 37.97968 |  |  |  |  |
|  | 54 | Nematodes | 1.585448 | 1.046155 | 2.770298 | 0.912452 | 2.770298 |
|  | 55 | Cestodes | 1.203291 | 0.822603 | 1.94586 | 0.743819 | 1.94586 |
|  | 13 | Trematodes | 10.8495 | 5.671955 | 25.03915 | 4.927675 | 25.03915 |
| Male  (n = 72) | 37 | *Trichostrongylus tenuis* | 2.171734 | 1.302055 | 3.997746 | 1.115294 | 3.997746 |
|  | 25 | *Heterakis dispar* | 2.404095 | 1.433277 | 4.548188 | 1.173052 | 4.548188 |
|  | 11 | *Amidostomum anseris* | 7.987238 | 3.977721 | 20.37579 | 3.361331 | 20.37579 |
|  | 2 | *Amidostomum spatulatum* | 33.12903 |  |  |  |  |
|  | 3 | *Epomidiostomum crami* | 28.61101 |  |  |  |  |
|  | 5 | *Tetrameres striata* | 36.15699 |  |  |  |  |
|  | 44 | *Tschertkovilepis setigera* | 1.809674 | 1.140583 | 3.13994 | 1.024483 | 3.13994 |
|  | 14 | *Drepanidotaenia lanceolata* | 5.252894 | 2.922804 | 11.1262 | 2.483768 | 11.1262 |
|  | 5 | *Wardoides nyrocae* | 25.27929 |  |  |  |  |
|  | 7 | *Notocotylus attenuatus* | 19.502 |  |  |  |  |
|  | 3 | U. trematode | 31.56022 |  |  |  |  |
|  | 42 | Nematodes | 1.278834 | 0.813754 | 2.355135 | 0.707162 | 2.355135 |
|  | 44 | Cestodes | 1.453322 | 0.946923 | 2.636839 | 0.813248 | 2.636839 |
|  | 9 | Trematodes | 15.44821 |  |  |  |  |
| Female  (n = 69) | 62 | *Trichostrongylus tenuis* | 1.28215 | 0.888111 | 2.058122 | 0.775755 | 2.058122 |
|  | 46 | *Heterakis dispar* | 2.788815 | 1.703767 | 5.072101 | 1.455915 | 5.072101 |
|  | 18 | *Amidostomum anseris* | 8.746988 | 4.768538 | 17.80102 | 4.032775 | 17.80102 |
|  | 4 | *Amidostomum spatulatum* | 10.45848 |  |  |  |  |
|  | 3 | *Epomidiostomum crami* | 26.96082 |  |  |  |  |
|  | 10 | *Tetrameres striata* | 9.396027 | 4.022426 | 24.3626 | 3.07432 | 24.3626 |
|  | 60 | *Tschertkovilepis setigera* | 1.377584 | 0.958004 | 2.203587 | 0.8506 | 2.203587 |
|  | 35 | *Drepanidotaenia lanceolata* | 6.416816 | 3.772218 | 12.37762 | 3.308462 | 12.37762 |
|  | 11 | *Wardoides nyrocae* | 15.91239 | 8.220387 | 39.83913 | 6.900424 | 39.83913 |
|  | 6 | *Notocotylus attenuatus* | 29.44392 | 14.18911 | 61.01559 | 11.31332 | 61.01559 |
|  | 0 | U. trematode |  |  |  |  |  |
|  | 68 | Nematodes | 0.71393 | 0.514798 | 1.110528 | 0.450915 | 1.110528 |
|  | 64 | Cestodes | 1.072356 | 0.765775 | 1.662633 | 0.688069 | 1.662633 |
|  | 7 | Trematodes | 29.05733 | 13.68616 | 63.65241 | 10.75567 | 63.65241 |
| Arctic  (n = 99) | 88 | *Trichostrongylus tenuis* | 1.435385 | 1.03558 | 2.237559 | 0.933506 | 2.237559 |
|  | 57 | *Heterakis dispar* | 2.675173 | 1.854596 | 4.720429 | 1.684376 | 4.720429 |
|  | 0 | *Amidostomum anseris* |  |  |  |  |  |
|  | 0 | *Amidostomum spatulatum* |  |  |  |  |  |
|  | 0 | *Epomidiostomum crami* |  |  |  |  |  |
|  | 14 | *Tetrameres striata* | 55.08323 | 27.22869 | 106.1598 | 19.39862 | 106.1598 |
|  | 96 | *Tschertkovilepis setigera* | 1.410833 | 1.02982 | 2.153971 | 0.931917 | 2.153971 |
|  | 41 | *Drepanidotaenia lanceolata* | 4.785929 | 3.169832 | 8.536333 | 2.856707 | 8.536333 |
|  | 10 | *Wardoides nyrocae* | 29.7153 | 14.462 | 72.26833 | 11.67164 | 72.26833 |
|  | 13 | *Notocotylus attenuatus* | 20.8233 | 10.77707 | 46.50513 | 9.31199 | 46.50513 |
|  | 0 | U. trematode |  |  |  |  |  |
|  | 94 | Nematodes | 1.011573 | 0.757877 | 1.474477 | 0.691924 | 1.474477 |
|  | 96 | Cestodes | 1.028485 | 0.768263 | 1.483176 | 0.691098 | 1.483176 |
|  | 16 | Trematodes | 18.34324 | 10.0876 | 38.71883 | 8.548474 | 38.71883 |
| Subarctic  (n = 42) | 31 | *Trichostrongylus tenuis* | 2.690331 | 1.604575 | 5.348173 | 1.403063 | 5.348173 |
|  | 33 | *Heterakis dispar* | 3.005655 | 1.780061 | 6.246053 | 1.509549 | 6.246053 |
|  | 14 | *Amidostomum anseris* | 7.257196 | 3.703312 | 16.82637 | 3.011005 | 16.82637 |
|  | 0 | *Amidostomum spatulatum* |  |  |  |  |  |
|  | 0 | *Epomidiostomum crami* |  |  |  |  |  |
|  | 3 | *Tetrameres striata* | 16.46535 |  |  |  |  |
|  | 27 | *Tschertkovilepis setigera* | 3.477996 | 2.040021 | 7.340567 | 1.733466 | 7.340567 |
|  | 25 | *Drepanidotaenia lanceolata* | 13.86131 | 6.576671 | 30.38797 | 5.531256 | 30.38797 |
|  | 16 | *Wardoides nyrocae* | 4.320196 | 2.342248 | 9.575247 | 2.065335 | 9.575247 |
|  | 3 | *Notocotylus attenuatus* | 9.376471 |  |  |  |  |
|  | 0 | U. trematode |  |  |  |  |  |
|  | 42 | Nematodes | 1.013839 | 0.655309 | 1.775724 | 0.56267 | 1.775724 |
|  | 35 | Cestodes | 3.619636 | 2.054891 | 6.938569 | 1.815621 | 6.938569 |
|  | 4 | Trematodes | 4.555906 |  |  |  |  |

**Table B6.** Pearson product-moment correlations for helminth group prevalence (top diagonal) and infection intensity (bottom diagnonal) for 141 samples of black brant (BLBR) and greater white fronted geese (GWFG) in Alaska. Samples are combined (OVERALL), and split into host species (GWFG and BLBR), location (Subarctic or Arctic), and host species by location (Arctic BLBR). Bold denotes correlations with paired t-test results with p < 0.15. Missing values in columns represent groups that had 100% prevalence and thus no standard deviation.

|  |  | **Nematodes** | **Cestodes** | **Trematodes** |
| --- | --- | --- | --- | --- |
| Overall |  |  |  |  |
|  | Nematodes |  | -0.05298 | -0.03196 |
|  | Cestodes | **-0.17657** |  | 0.033137 |
|  | Trematodes | -0.11169 | -0.00804 |  |
| GWFG |  |  |  |  |
|  | Nematodes | 1 | -0.06356 | -0.01688 |
|  | Cestodes | **-0.2649** | 1 | -0.06116 |
|  | Trematodes | -0.1376 | -0.07996 | 1 |
| BLBR |  |  |  |  |
|  | Nematodes |  | -0.03352 | 0.033515 |
|  | Cestodes | -0.0139 |  | 0.092105 |
|  | Trematodes | **0.17679** | -0.00951 |  |
| Arctic BLBR |  |  |  |  |
|  | Nematodes |  | -- | 0.044426 |
|  | Cestodes | **-0.30542** |  | -- |
|  | Trematodes | **0.52880^a^** | -0.1820501 |  |
| Arctic |  |  |  |  |
|  | Nematodes |  | -0.04077 | -0.02403 |
|  | Cestodes | **-0.28032** |  | -0.08247 |
|  | Trematodes | -0.12881 | -0.0511 |  |
| Subarctic |  |  |  |  |
|  | Nematodes |  | -- | -- |
|  | Cestodes | 0.14729 |  | 0.145095 |
|  | Trematodes | **-0.18748** | **0.17997** |  |

^a^Only 3 Arctic brant were infected with trematodes and thus, we suggest caution when interpreting this correlation.

**Table B7.** Pearson product-moment correlations (top diagonal) and paired *t*-test p-values (lower diagonal) for prevalence of helminth species identified in 141 samples of black brant (BLBR) and greater white fronted geese (GWFG) in Alaska. Samples are combined (OVERALL), and split into species (GWFG and TOTAL BLBR), location (SUBARCTIC or ARCTIC) and location by species for black brant (ARCTIC BLBR). Bold denotes correlations and test results with p < 0.15. Missing values in columns represent species that were either absent from the sample (e.g., *E. crami* in Subarctic brant) or had 100% prevalence and thus no standard deviation.

| **OVERALL** | *T. tenuis* | *H. dispar* | *A. anseris* | *A. spatulatum* | *E. crami* | *T. striata* | *T. setigera* | *D. lanceolata* | *W. nyrocae* | *N. attenuatus* | *U. trematode* |
| --- | --- | --- | --- | --- | --- | --- | --- | --- | --- | --- | --- |
| *T. tenuis* | 1.000 | 0.083 | 0.028 | 0.008 | -0.006 | -0.021 | 0.070 | 0.129 | 0.003 | -0.031 | 0.082 |
| *H. dispar* | 0.327 | 1.000 | -0.169 | -0.032 | -0.134 | -0.084 | -0.111 | 0.262 | 0.053 | -0.243 | -0.015 |
| *A. anseris* | 0.745 | 0.046 | 1.000 | 0.091 | 0.279 | 0.033 | -0.068 | -0.158 | 0.015 | 0.149 | 0.152 |
| *A. spatulatum* | 0.922 | 0.708 | 0.284 | 1.000 | -0.048 | 0.116 | 0.087 | -0.084 | -0.024 | -0.082 | 0.133 |
| *E. crami* | 0.942 | 0.114 | 0.001 | 0.570 | 1.000 | -0.078 | 0.081 | -0.198 | -0.100 | -0.075 | -0.040 |
| *T. striata* | 0.806 | 0.323 | 0.698 | 0.171 | 0.358 | 1.000 | 0.011 | 0.002 | 0.105 | 0.074 | -0.071 |
| *T. setigera* | 0.411 | 0.190 | 0.420 | 0.303 | 0.342 | 0.896 | 1.000 | 0.061 | 0.072 | 0.070 | 0.073 |
| *D. lanceolata* | 0.127 | 0.002 | 0.061 | 0.325 | 0.019 | 0.983 | 0.474 | 1.000 | 0.214 | -0.156 | -0.026 |
| *W. nyrocae* | 0.973 | 0.529 | 0.858 | 0.773 | 0.237 | 0.216 | 0.394 | 0.011 | 1.000 | 0.003 | 0.008 |
| *N. attenuatus* | 0.715 | 0.004 | 0.077 | 0.335 | 0.374 | 0.386 | 0.410 | 0.064 | 0.973 | 1.000 | 0.052 |
| *U. trematode* | 0.331 | 0.857 | 0.073 | 0.117 | 0.634 | 0.403 | 0.387 | 0.758 | 0.928 | 0.538 | 1.000 |

| **SUBARCTIC** | *T. tenuis* | *H. dispar* | *A. anseris* | *A. spatulatum* | *E. crami* | *T. striata* | *T. setigera* | *D. lanceolata* | *W. nyrocae* | *N. attenuatus* | *U. trematode* |
| --- | --- | --- | --- | --- | --- | --- | --- | --- | --- | --- | --- |
| *T. tenuis* | 1.000 | -0.047 | 0.077 | 0.093 |  | -0.045 | -0.105 | 0.391 | 0.133 | -0.045 | 0.093 |
| *H. dispar* | 0.767 | 1.000 | 0.000 | 0.082 |  | -0.531 | -0.268 | -0.194 | -0.307 | -0.306 | 0.082 |
| *A. anseris* | 0.630 | 1.000 | 1.000 | -0.110 |  | -0.196 | 0.000 | 0.069 | -0.035 | 0.392 | -0.110 |
| *A. spatulatum* | 0.558 | 0.608 | 0.486 | 1.000 |  | -0.043 | 0.116 | 0.129 | -0.123 | -0.043 | -0.024 |
| *E. crami* |  |  |  |  |  |  |  |  |  |  |  |
| *T. striata* | 0.777 | 0.000 | 0.213 | 0.785 |  | 1.000 | 0.207 | 0.040 | -0.027 | -0.077 | -0.043 |
| *T. setigera* | 0.508 | 0.086 | 1.000 | 0.463 |  | 0.189 | 1.000 | 0.195 | 0.380 | 0.207 | 0.116 |
| *D. lanceolata* | 0.010 | 0.218 | 0.666 | 0.416 |  | 0.800 | 0.215 | 1.000 | 0.247 | 0.229 | 0.129 |
| *W. nyrocae* | 0.402 | 0.048 | 0.827 | 0.440 |  | 0.864 | 0.013 | 0.114 | 1.000 | 0.163 | 0.199 |
| *N. attenuatus* | 0.777 | 0.049 | 0.010 | 0.785 |  | 0.628 | 0.189 | 0.145 | 0.302 | 1.000 | -0.043 |
| *U. trematode* | 0.558 | 0.608 | 0.486 | 0.878 |  | 0.785 | 0.463 | 0.416 | 0.206 | 0.785 | 1.000 |

|  |  |  |  |  |  |  |  |  |  |  |  |
| --- | --- | --- | --- | --- | --- | --- | --- | --- | --- | --- | --- |
| **ARCTIC** | *T. tenuis* | *H. dispar* | *A. anseris* | *A. spatulatum* | *E. crami* | *T. striata* | *T. setigera* | *D. lanceolata* | *W. nyrocae* | *N. attenuatus* | *U. trematode* |
| *T. tenuis* | 1.000 | 0.217 | 0.034 | -0.045 | -0.045 | -0.041 | 0.125 | 0.036 | 0.012 | -0.053 | 0.073 |
| *H. dispar* | 0.031 | 1.000 | -0.279 | -0.039 | -0.125 | 0.055 | 0.206 | 0.390 | 0.152 | -0.211 | -0.031 |
| *A. anseris* | 0.736 | 0.005 | 1.000 | 0.170 | 0.373 | 0.132 | -0.047 | -0.301 | -0.018 | 0.080 | 0.260 |
| *A. spatulatum* | 0.659 | 0.702 | 0.093 | 1.000 | -0.065 | 0.140 | 0.045 | -0.128 | 0.055 | -0.099 | 0.163 |
| *E. crami* | 0.659 | 0.219 | 0.000 | 0.526 | 1.000 | -0.103 | 0.045 | -0.214 | -0.085 | -0.099 | -0.052 |
| *T. striata* | 0.687 | 0.588 | 0.194 | 0.167 | 0.310 | 1.000 | -0.266 | 0.012 | 0.249 | 0.100 | -0.083 |
| *T. setigera* | 0.218 | 0.041 | 0.642 | 0.659 | 0.659 | 0.008 | 1.000 | 0.149 | 0.059 | -0.106 | 0.036 |
| *D. lanceolata* | 0.722 | 0.000 | 0.002 | 0.208 | 0.034 | 0.907 | 0.142 | 1.000 | 0.126 | -0.266 | -0.068 |
| *W. nyrocae* | 0.907 | 0.133 | 0.860 | 0.586 | 0.402 | 0.013 | 0.560 | 0.212 | 1.000 | -0.031 | -0.069 |
| *N. attenuatus* | 0.603 | 0.036 | 0.432 | 0.331 | 0.331 | 0.326 | 0.298 | 0.008 | 0.760 | 1.000 | 0.072 |
| *U. trematode* | 0.475 | 0.757 | 0.009 | 0.107 | 0.608 | 0.413 | 0.722 | 0.501 | 0.499 | 0.478 | 1.000 |
|  |  |  |  |  |  |  |  |  |  |  |  |
| **GWFG** | *T. tenuis* | *H. dispar* | *A. anseris* | *A. spatulatum* | *E. crami* | *T. striata* | *T. setigera* | *D. lanceolata* | *W. nyrocae* | *N. attenuatus* | *U. trematode* |
| *T. tenuis* | 1.000 | 0.185 | 0.080 | -0.055 | -0.055 | -0.028 | 0.132 | 0.008 | 0.123 | -0.082 | 0.109 |
| *H. dispar* | 0.164 | 1.000 | -0.145 | 0.165 | -0.094 | -0.008 | 0.169 | 0.234 | 0.294 | -0.139 | 0.089 |
| *A. anseris* | 0.552 | 0.278 | 1.000 | 0.165 | 0.294 | 0.230 | 0.006 | -0.180 | 0.036 | -0.043 | 0.232 |
| *A. spatulatum* | 0.680 | 0.216 | 0.216 | 1.000 | -0.094 | 0.299 | 0.072 | -0.041 | 0.125 | -0.140 | 0.159 |
| *E. crami* | 0.680 | 0.485 | 0.025 | 0.481 | 1.000 | -0.104 | 0.072 | -0.181 | -0.094 | -0.140 | -0.084 |
| *T. striata* | 0.833 | 0.951 | 0.082 | 0.023 | 0.436 | 1.000 | -0.432 | -0.201 | 0.299 | 0.145 | -0.092 |
| *T. setigera* | 0.322 | 0.204 | 0.966 | 0.593 | 0.593 | 0.001 | 1.000 | 0.138 | 0.072 | -0.099 | 0.064 |
| *D. lanceolata* | 0.953 | 0.077 | 0.176 | 0.759 | 0.173 | 0.131 | 0.302 | 1.000 | 0.099 | -0.165 | -0.005 |
| *W. nyrocae* | 0.358 | 0.025 | 0.790 | 0.352 | 0.481 | 0.023 | 0.593 | 0.459 | 1.000 | 0.022 | -0.084 |
| *N. attenuatus* | 0.540 | 0.298 | 0.748 | 0.294 | 0.294 | 0.278 | 0.457 | 0.215 | 0.867 | 1.000 | 0.056 |
| *U. trematode* | 0.416 | 0.507 | 0.080 | 0.234 | 0.533 | 0.490 | 0.635 | 0.968 | 0.533 | 0.677 | 1.000 |

|  |  |  |  |  |  |  |  |  |  |  |  |
| --- | --- | --- | --- | --- | --- | --- | --- | --- | --- | --- | --- |
| **ARCTIC BLBR** | *T. tenuis* | *H. dispar* | *A. anseris* | *A. spatulatum* | *E. crami* | *T. striata* | *T. setigera* | *D. lanceolata* | *W. nyrocae* | *N. attenuatus* | *U. trematode* |
| *T. tenuis* | 1.000 | 0.223 | 0.064 | 0.044 | 0.044 | -0.098 |  | -0.019 | -0.182 | 0.079 |  |
| *H. dispar* | 0.161 | 1.000 | 0.074 | -0.481 | 0.052 | -0.046 |  | 0.262 | -0.129 | -0.223 |  |
| *A. anseris* | 0.693 | 0.644 | 1.000 | -0.036 | 0.698 | 0.174 |  | -0.298 | -0.084 | 0.371 |  |
| *A. spatulatum* | 0.783 | 0.001 | 0.824 | 1.000 | -0.025 | -0.078 |  | -0.208 | -0.059 | -0.044 |  |
| *E. crami* | 0.783 | 0.747 | 0.000 | 0.877 | 1.000 | -0.078 |  | -0.208 | -0.059 | -0.044 |  |
| *T. striata* | 0.542 | 0.777 | 0.276 | 0.629 | 0.629 | 1.000 |  | 0.118 | 0.193 | 0.098 |  |
| *T. setigera* |  |  |  |  |  |  |  |  |  |  |  |
| *D. lanceolata* | 0.906 | 0.098 | 0.058 | 0.192 | 0.192 | 0.461 |  | 1.000 | 0.128 | -0.370 |  |
| *W. nyrocae* | 0.256 | 0.423 | 0.600 | 0.714 | 0.714 | 0.227 |  | 0.424 | 1.000 | -0.105 |  |
| *N. attenuatus* | 0.624 | 0.161 | 0.017 | 0.783 | 0.783 | 0.542 |  | 0.017 | 0.515 | 1.000 |  |
| *U. trematode* |  |  |  |  |  |  |  |  |  |  |  |
|  |  |  |  |  |  |  |  |  |  |  |  |
| **TOTAL BLBR** | *T. tenuis* | *H. dispar* | *A. anseris* | *A. spatulatum* | *E. crami* | *T. striata* | *T. setigera* | *D. lanceolata* | *W. nyrocae* | *N. attenuatus* | *U. trematode* |
| *T. tenuis* | 1.000 | 0.071 | -0.025 | 0.071 | 0.050 | -0.014 | 0.039 | 0.238 | -0.034 | 0.001 | 0.050 |
| *H. dispar* | 0.521 | 1.000 | -0.042 | -0.148 | 0.048 | -0.223 | -0.116 | -0.001 | -0.283 | -0.264 | 0.048 |
| *A. anseris* | 0.826 | 0.709 | 1.000 | -0.077 | 0.226 | -0.101 | -0.167 | -0.052 | 0.067 | 0.335 | -0.054 |
| *A. spatulatum* | 0.525 | 0.180 | 0.490 | 1.000 | -0.017 | -0.061 | 0.074 | -0.037 | -0.091 | -0.044 | -0.017 |
| *E. crami* | 0.655 | 0.669 | 0.040 | 0.876 | 1.000 | -0.043 | 0.052 | -0.139 | -0.064 | -0.031 | -0.012 |
| *T. striata* | 0.902 | 0.043 | 0.364 | 0.581 | 0.698 | 1.000 | 0.184 | 0.091 | 0.018 | 0.028 | -0.043 |
| *T. setigera* | 0.724 | 0.295 | 0.130 | 0.507 | 0.641 | 0.097 | 1.000 | 0.143 | 0.129 | 0.131 | 0.052 |
| *D. lanceolata* | 0.030 | 0.994 | 0.640 | 0.740 | 0.209 | 0.415 | 0.198 | 1.000 | 0.176 | -0.066 | 0.087 |
| *W. nyrocae* | 0.761 | 0.010 | 0.548 | 0.411 | 0.564 | 0.874 | 0.244 | 0.111 | 1.000 | 0.052 | 0.190 |
| *N. attenuatus* | 0.989 | 0.016 | 0.002 | 0.694 | 0.782 | 0.801 | 0.237 | 0.555 | 0.643 | 1.000 | -0.031 |
| *U. trematode* | 0.655 | 0.669 | 0.628 | 0.876 | 0.913 | 0.698 | 0.641 | 0.432 | 0.086 | 0.782 | 1.000 |


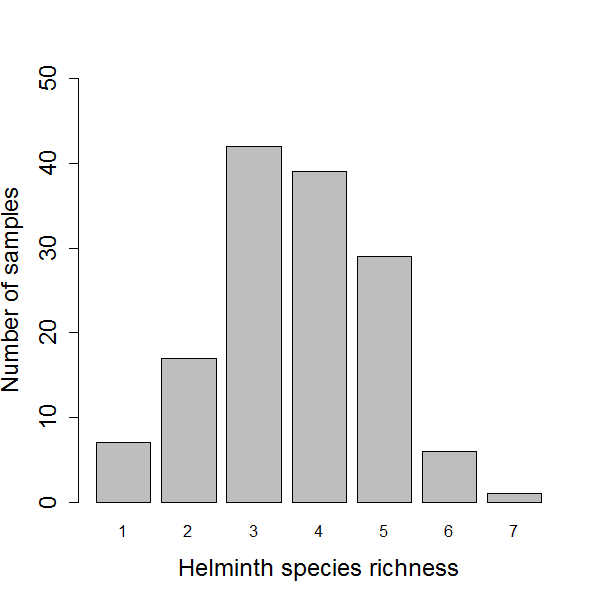


**Fig. B1.** Frequency distribution of helminth species richness for Pacific black brant and greater white-fronted geese collected in Alaska, 2014.


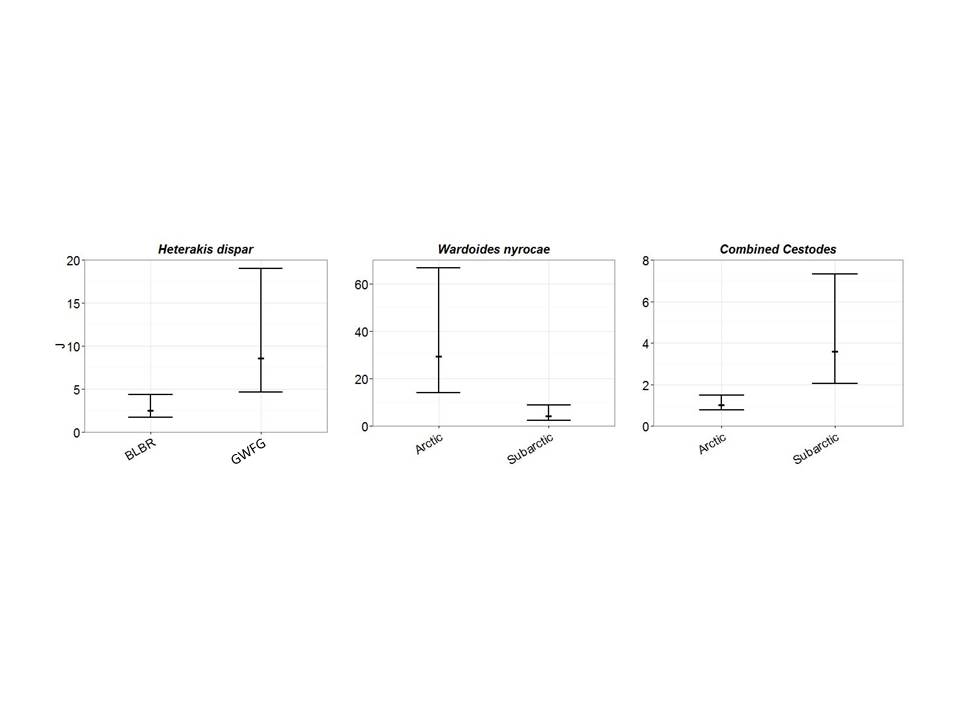


**Fig. B2.** Boulinier’s *J* index of parasite aggregation (short horizontal bar) for all samples of Pacific black brant (BLBR; n = 83) and greater white-fronted geese (GWFG; n = 58) collected in Subarctic and Arctic Alaska, 2014. Groups presented had 85% confidence intervals that did not overlap for a given parasite species or class, suggesting differences in parasite aggregations among groups. Error bars denote 85% confidence intervals derived by bootstrapping simulated distributions of *J* (Sherrard-Smith et al. 2015).


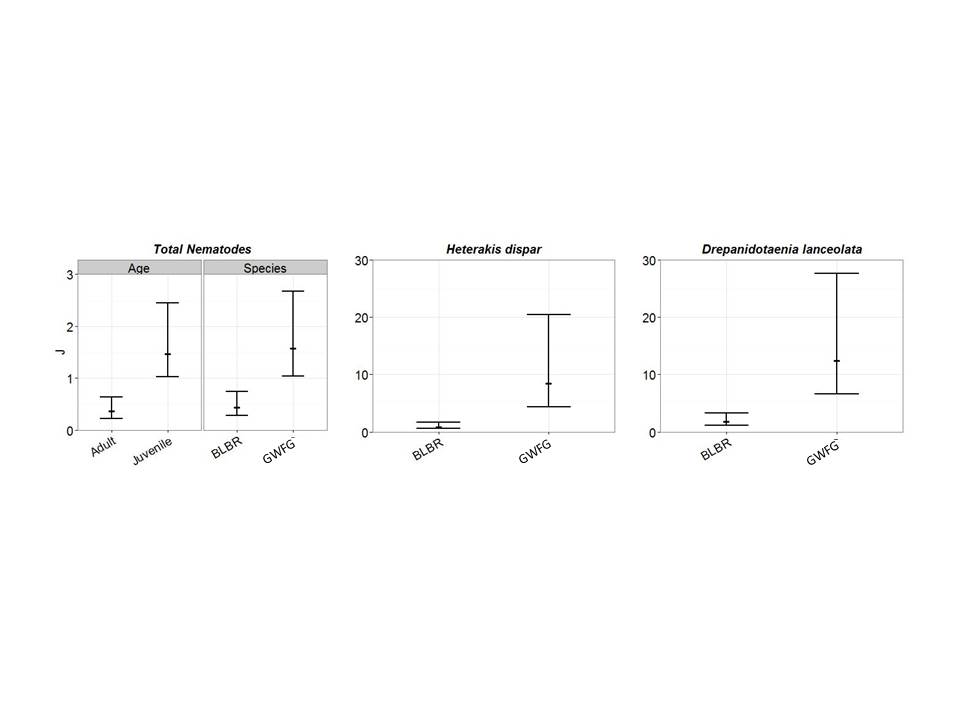


**Fig. B3.** Boulinier’s *J* index of parasite aggregation (short horizontal bar) for Arctic-only Pacific black brant (BLBR; n = 41) and greater white-fronted geese (GWFG; n = 58) collected in Alaska, 2014. Groups presented (i.e., age class, adult or juvenile, and species) had 85% confidence intervals that did not overlap for a given parasite species or class, suggesting differences in parasite aggregations among groups. Error bars denote 85% confidence intervals derived by bootstrapping simulated distributions of *J* (Sherrard-Smith et al. 2015).


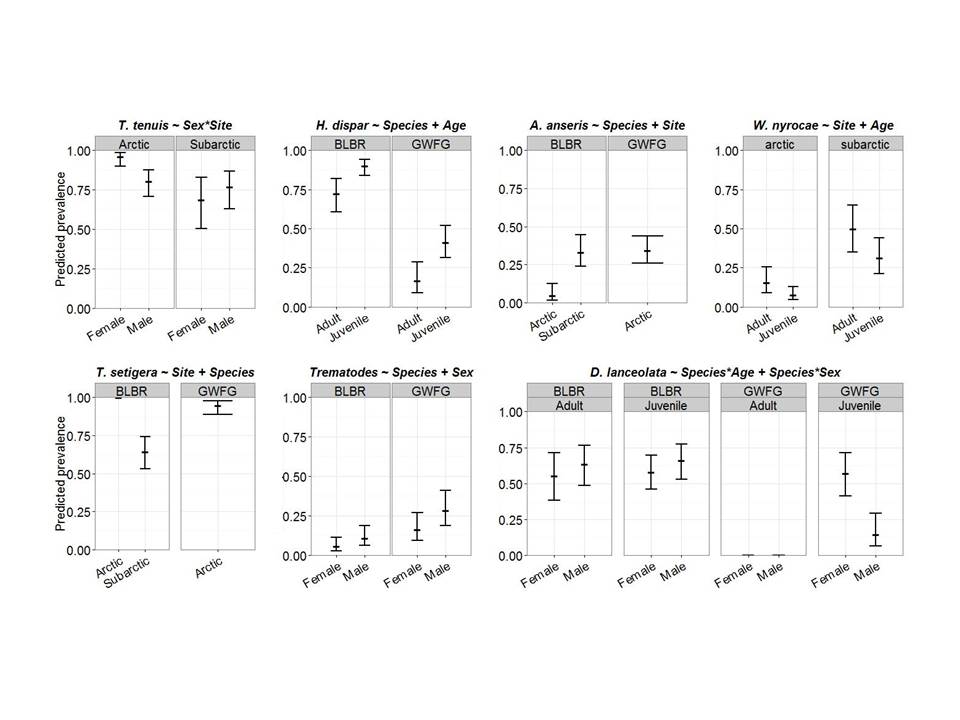


**Fig. B4.** Predicted helminth prevalence for all trematodes (i.e., *N. attenuatus* and unidentified spp.), three nematodes (*H. dispar, A. anseris, and T. tenuis*) and one cestode (*D. lanceolata*) identified in black brant (BLBR) and greater white-fronted geese (GWFG) collected in Subarctic and Arctic Alaska, 2014. Short dashes represent predicted means and error bars denote 85% confidence intervals. Results are based off the most supported models from AIC_c_ selection (see titles).


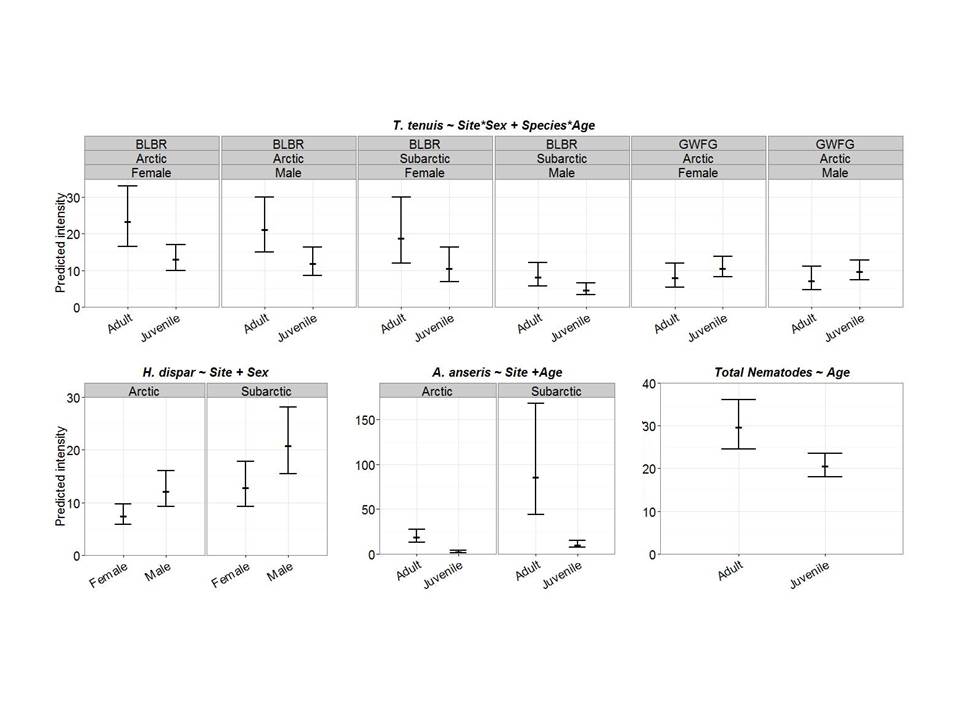


**Fig. B5.** Predicted helminth infection intensity for combined nematodes, and three nematode species identified in black brant (BLBR) and greater white-fronted geese (GWFG) collected in two locations (i.e., Arctic and Subarctic) in Alaska, 2014. Short dashes represent predicted means and error bars denote 85% confidence intervals. Results are based off the most supported models from AIC_c_ selection (see titles).


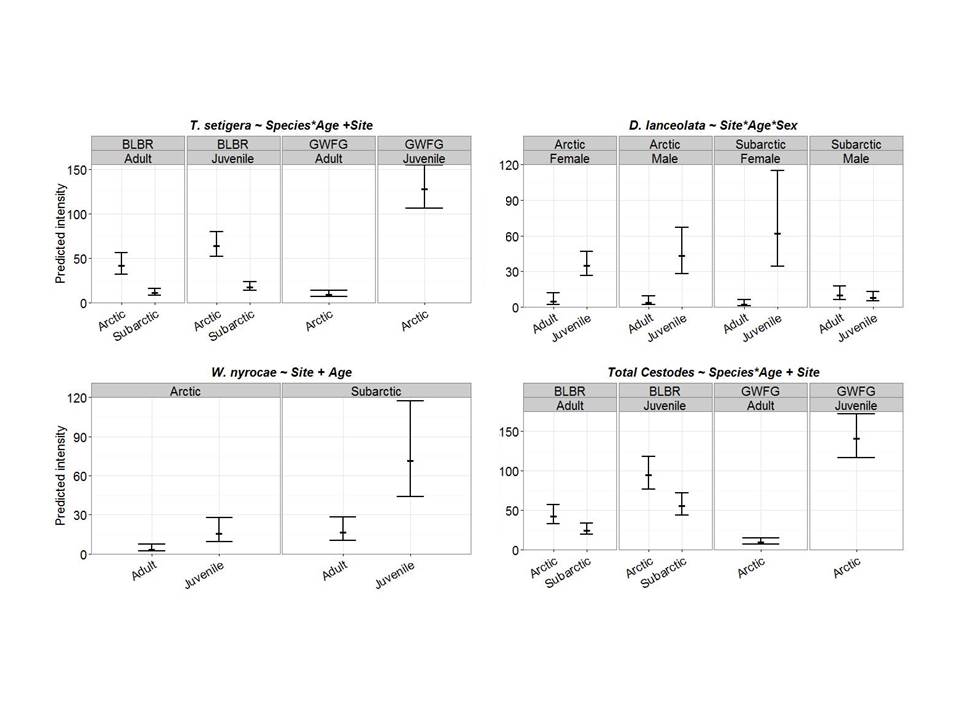


**Fig. B6.**  Predicted helminth infection intensity for combined cestodes, and three cestode species identified in black brant (BLBR) and greater white-fronted geese (GWFG) collected in two locations (i.e., Arctic and Subarctic) in Alaska, 2014. Short dashes represent predicted means and error bars denote 85% confidence intervals. Results are based off the most supported models from AIC_c_ selection (see titles).
